# Altered oculomotor signatures of emotional conflict and valence processing in late-life depression

**DOI:** 10.1016/j.ijchp.2026.100711

**Published:** 2026-07-31

**Authors:** Yu-Ming Wu, Yao-Tung Lee, Yi-Hsuan Chang, Li-Kai Huang, Kuan-Yu Chen, Chaowen Chen, Ta-Liang Chen, Chin-An Wang

**Affiliations:** a Department of Anesthesiology, School of Medicine, College of Medicine, Taipei Medical University, Taipei, Taiwan; b Department of Anesthesiology, Shuang Ho Hospital, Taipei Medical University, Taipei, Taiwan; c Department of Psychiatry, Shuang Ho Hospital, Taipei Medical University, Taipei, Taiwan; d Department of Psychiatry, School of Medicine, College of Medicine, Taipei Medical University, Taipei, Taiwan; e Eye-Tracking Laboratory, Shuang Ho Hospital, Taipei Medical University, Taipei, Taiwan; f Institute of Cognitive Neuroscience, National Central University, Taoyuan, Taiwan; g Department of Neurology, School of Medicine, College of Medicine, Taipei Medical University, Taipei, Taiwan; h Dementia Center and Department of Neurology, Shuang Ho Hospital, Taipei Medical University, Taipei, Taiwan; i Department of Psychiatry and Behavioral Sciences, University of Texas Health Science Center, Houston, TX, United States; j Health Policy Research Center, Taipei Medical University Hospital, Taipei, Taiwan; k Department of Anesthesiology, Wang Fang Hospital, Taipei Medical University, Taipei, Taiwan; l Department of Education and Humanities in Medicine, School of Medicine, College of Medicine, Taipei Medical University, Taipei, Taiwan; m Ph.D. Program in Medical Neuroscience, College of Medical Science and Technology, Taipei Medical University, Taipei, Taiwan

**Keywords:** Emotional interference, Fixational eye movements, Microsaccades, Superior colliculus, Locus coeruleus, Eye-tracking

## Abstract

Late-life depression (LLD) is a heterogeneous neuropsychiatric condition characterized by affective, cognitive, and autonomic disturbances, underscoring the need for objective markers that index dysfunction in specific neural control systems. Microsaccades, small fixational eye movements generated through oculomotor circuitry that includes the superior colliculus, provide a temporally precise behavioral measure sensitive to arousal and cognitive control signals, yet their clinical relevance in LLD remains largely unexplored. In this study, we examined whether task-evoked microsaccade dynamics are altered in LLD during emotional conflict and valence processing using an emotional face–word Stroop task. Microsaccade rate and kinematic metrics were analyzed in 26 individuals with LLD and 27 healthy older adults (CTRL). Across groups, microsaccade rates were higher during emotionally incongruent compared with congruent trials, indicating sensitivity to emotional conflict, with a trend toward lower overall rates in LLD. During valence processing, microsaccade rates were higher for fearful than happy faces and were significantly lower in LLD than in CTRL participants. In contrast, microsaccade kinematics were largely preserved across groups. Exploratory inter-individual analyses identified nominal associations with somatic symptom burden and cognitive status, but none survived correction for multiple comparisons. An exploratory multivariate classification analysis combining microsaccade, pupil, eye-blink, and reaction time indices yielded chance-level differentiation between groups. Together, these findings indicate that microsaccade rate and kinematics are sensitive to emotional conflict and valence processing, with selective alterations in these dynamics in LLD, highlighting microsaccades as a potentially informative behavioral index of affective interference control in clinical aging.

## Introduction

Late-life depression (LLD) refers to major depressive disorder occurring in older adulthood and represents a prevalent and clinically significant condition associated with increased disability, medical comorbidity, and mortality (Alexopoulos, 2019; Aziz & Steffens, 2013; Djernes, 2006; Tang et al., 2022). LLD is a heterogeneous neuropsychiatric disorder (Jellinger, 2023) characterized by a broad constellation of affective, cognitive, autonomic, and somatic disturbances (Adriani et al., 2024; Penninx et al., 2013). In LLD, these depressive symptoms often coexist with executive dysfunction (Rajtar-Zembaty et al., 2017), emotional dysregulation (Szymkowicz et al., 2023), attention deficits (Lockwood et al., 2002), and prominent physical complaints (Van Damme et al., 2018), making the clinical presentation highly variable and often difficult to disentangle using symptom-based assessments alone (Koenig et al., 2014). This heterogeneity has motivated growing interest in identifying objective, neurobiologically grounded markers (Alexopoulos, 2019) that can capture dysfunction in specific brain networks implicated in LLD, rather than treating the disorder as a unitary clinical entity (Drysdale et al., 2017).

One neural system that has received increasing attention in LLD is the locus coeruleus–norepinephrine (LC–NE) network (Aston-Jones & Cohen, 2005; Poe et al., 2020). The LC–NE system plays a central role in regulating arousal and cognitive control, and its structural and functional integrity is known to change with ageing (Aston-Jones & Cohen, 2005; Liu et al., 2020; Mather & Harley, 2016; Poe et al., 2020). Dysregulation of LC–NE signaling has been implicated in depression-related alterations (Bernard et al., 2011; Chan‐Palay & Asan, 1989; Du et al., 2020; Klimek et al., 1997; Llorca-Torralba et al., 2022), and research, primarily from imaging studies, has demonstrated disruptions in the LC-centered system in LLD compared to healthy controls (del Cerro et al., 2020; Guinea-Izquierdo et al., 2021; Tsopelas et al., 2011). While neuroimaging approaches provide valuable insight into these impairments, their cost and methodological complexity limit their widespread use for probing LC–NE function in large or diverse samples. These limitations underscore the need for accessible and theory-driven behavioral paradigms that can probe LC–NE–related executive and emotional control processes in older adults.

From a functional perspective, LC–NE system dysregulation in LLD is particularly evident in processes requiring the prioritization of emotionally salient information under conditions of competition (Mather et al., 2016). Because the LC–NE system contributes to neural gain and the prioritization of behaviorally relevant information (Aston-Jones & Cohen, 2005), emotional conflict control and valence processing provide behavioral contexts in which LC–NE-related influences may be engaged. The emotional face–word Stroop task (Etkin et al., 2006, 2011) is a well-validated paradigm for examining the resolution of competition between target facial expressions and incongruent emotional distractors. Neuroimaging and pupillometric studies have implicated LC–NE-related processes in emotional interference resolution and associated prefrontal and cingulate activity (Grueschow et al., 2020, 2021, 2022). These findings position the task as a useful framework for examining emotional control processes that may be influenced by LC–NE signaling. However, while it is established that congruency and valence modulate global behavioral and autonomic responses (Chang et al., 2023; Grueschow et al., 2021), how emotional congruency and valence influence fine-grained oculomotor behavior remains largely unexplored in LLD.

Microsaccades, small, fixation-related eye movements, provide a complementary and mechanistically informative index of oculomotor and attentional control processes (Contadini-Wright et al., 2023; Dalmaso et al., 2017, 2020; Engbert & Kliegl, 2003; Hafed & Clark, 2002; Hermens et al., 2010; Siegenthaler et al., 2014; Watanabe et al., 2013). Experimental evidence indicates that the superior colliculus (SC) plays a causal role in microsaccade generation (Hafed et al., 2009, 2013). The SC is a midbrain structure that integrates sensory, cognitive, and arousal-related signals to guide orienting behavior (Basso & May 2017; Corneil & Munoz, 2014; Chin-An Wang & Munoz, 2024; White & Munoz, 2011). Importantly, the SC receives direct anatomical projections from regions implicated in emotional conflict and valence processing, including the LC (Edwards et al., 1979; Li et al., 2018), ACC (Leichnetz et al., 1981), and dorsolateral prefrontal cortex (DLPFC) (Goldman & Nauta, 1976), providing a neuroanatomical substrate through which emotional and control signals can influence microsaccade generation. Consistent with this framework, recent work in healthy young adults has shown that microsaccade dynamics are modulated by emotional processing in the emotional face–word Stroop task (Chang et al., 2025), in line with broader evidence linking microsaccades to emotion and conflict processing (Dalmaso et al., 2019; Krejtz et al., 2020). However, whether such microsaccade signatures are altered in older adults with clinically diagnosed late-life depression remains unknown. Critically, it is also unclear whether emotional conflict and emotional valence exert distinct effects on microsaccade behavior in LLD, and whether these effects relate to inter-individual differences in symptom severity or cognitive status. Addressing these questions would extend current understanding of LLD by characterizing a temporally precise oculomotor output generated by circuitry including the SC.

In the present study, we investigated task-evoked microsaccade dynamics in older adults with LLD and age-matched healthy controls (CTRL) performing the emotional face–word Stroop task. We focused on microsaccade rate and kinematic measures and quantified modulation indices for emotional congruency (incongruent minus congruent) and emotional valence (fear minus happy). Guided by the established anatomical connections of the SC with the LC, ACC, and DLPFC, and by prior evidence linking microsaccades to emotional processing, the present study tested the following a priori predictions. First, we predicted that emotional conflict would modulate microsaccade behavior, such that incongruent trials would elicit altered microsaccade rates and kinematics relative to congruent trials, reflecting increased arousal and control demands. Second, given evidence for LC–NE involvement in processing emotional valenced stimuli, we predicted that emotional valence would similarly influence microsaccade dynamics, with fearful expressions producing distinct modulation compared to happy expressions. Third, because LLD is associated with alterations in arousal and cognitive-control processes, to which LC–NE signaling may contribute, we predicted that these conflict- and valence-related microsaccade modulations would differ between LLD and CTRL participants. Finally, we explored whether inter-individual variability in microsaccade modulation was related to depressive symptom severity, somatic symptom burden, and global cognitive function, providing insight into the clinical relevance of microsaccade-based indices of emotional control in late-life depression.

## Materials and methods

*Experimental setup.* All experimental procedures were reviewed and approved by the Institutional Review Board of Taipei Medical University, Taiwan, and were conducted in accordance with the Declaration of Helsinki (World Medical Association, 2001). Participants were naïve to the purpose of the experiment, provided written informed consent, and received monetary compensation. Initially, 35 individuals with LLD and 35 healthy older adults were recruited. Five participants were excluded because their response accuracy was below 20% in at least one task condition (CTRL: 3; LLD: 2), and one additional LLD participant was excluded because accuracy was below 50% in both task conditions. Eleven participants were excluded because a technical recording failure produced a constant vertical eye-position signal, which prevented valid binocular microsaccade detection (CTRL: 5; LLD: 6). The final sample therefore comprised twenty-six LLD patients (mean age = 71 years, range = 61–81 years) and twenty-seven healthy older adults (mean age = 73 years, range = 65–85 years). Each patient completed standardized assessments of cognitive function and clinical status, including the Montreal Cognitive Assessment (MoCA), the 15-item Geriatric Depression Scale (GDS-15), and the somatic symptom subscale of the Patient Health Questionnaire (PHQ-15) (De Craen et al., 2003; Kroenke et al., 2002; Nasreddine et al., 2005), using validated Chinese versions (Lee et al., 2011; Liao et al., 1995; Tsai et al., 2012).

Eligibility criteria for the LLD group were: age ≥ 60 years; DSM-5 diagnosis of a nonpsychotic, unipolar major depressive episode; and first onset of depression at age ≥ 60 years. Diagnoses were confirmed independently by a board-certified geriatric psychiatrist (co-author YL) and a board-certified neurologist (co-author LH). Participants were required to be cognitively intact, with no clinical diagnosis of mild cognitive impairment or dementia. To further minimize comorbid cognitive disorders, inclusion required Mini-Mental State Examination (MMSE) scores of ≥ 24 for participants with > 6 years of education, ≥ 21 for those with 1–6 years of education, and ≥ 17 for those with no formal education (Folstein et al., 1975). Additional exclusion criteria for all participants included current or past psychiatric disorders other than depression, major neurological disease, history of significant brain injury, or a prior diagnosis of cognitive disorder.

Patients continued their prescribed medications throughout the study in accordance with IRB guidelines. Medication exposure at the time of assessment was categorized as selective serotonin reuptake inhibitor (SSRI), serotonin–norepinephrine reuptake inhibitor (SNRI), norepinephrine–dopamine reuptake inhibitor (NDRI), tricyclic antidepressant (TCA), monoamine oxidase inhibitor (MAOI), α-blocker, β-blocker, anticholinergic medication, antihistamine, or benzodiazepine. Medication categories were not mutually exclusive because participants could be taking medications from more than one class. Medication-class distributions for the LLD and CTRL groups are reported in Supplementary Table 1. The CTRL group comprised 27 healthy older adults with no history of major psychiatric or neurological disorders. CTRL participants were spouses or friends of LLD participants or community members recruited via advertisement. CTRL did not differ significantly from LLD in age, years of education, PHQ-15 scores, or MoCA scores. The GDS-15 was not completed by one LLD participant and five CTRL participants due to technical issues. Demographic and clinical characteristics are summarized in Table 1. Since there were no prior studies examining microsaccade behavior using this task in LLD, sample sizes were selected based on our previous microsaccade studies in healthy individuals and clinical populations (Chang et al., 2025; Chen et al., 2021; Lee, Tai et al., 2025).

**Table 1:** Demographics and clinical score of participants.

Group	Number of Participants	Age at time of Measurement	Sex (male)	Education (years)	GDS15	PHQ15	MoCA
LLD	26	71.3 ± 4.8	10	9.0 ± 3.9	9.9 ± 2.9	3.9 ± 4.2	19.9 ± 4.5
CTRL	27	73.0 ± 5.7	10	10.0 ± 4.0	1.3 ± 1.3	2.2 ± 2.5	20.8 ± 5.2
		p = 0.24	p = 0.92	p = 0.38	p < 0.01	p = 0.11	p = 0.50

LLD: late-life depression patients. CTRL: healthy older adults. GDS: Geriatric Depression Scale. PHQ: Patient Health Questionnaire. MoCA: Montreal Cognitive Assessment. p: p-value for differences between groups.

*Recording and Apparatus.* Participants were seated in a dark room, with their head stabilized in a chin and forehead rest, and their eye position and pupil size were measured with a video-based eye tracker (Eyelink-1000 plus, SR Research, Osgoode, ON, Canada) at a rate of 1000 Hz with binocular recording. Stimulus presentation and data acquisition were controlled by Eyelink Experiment Builder and Eyelink software. The stimuli were presented on an LCD monitor at a screen resolution of 1920 × 1080 pixels with a refresh rate of 60 Hz, subtending a viewing angle of 43° x 24°, with the distance from the eyes to the monitor set at 80 cm.

**Fig. 1:**
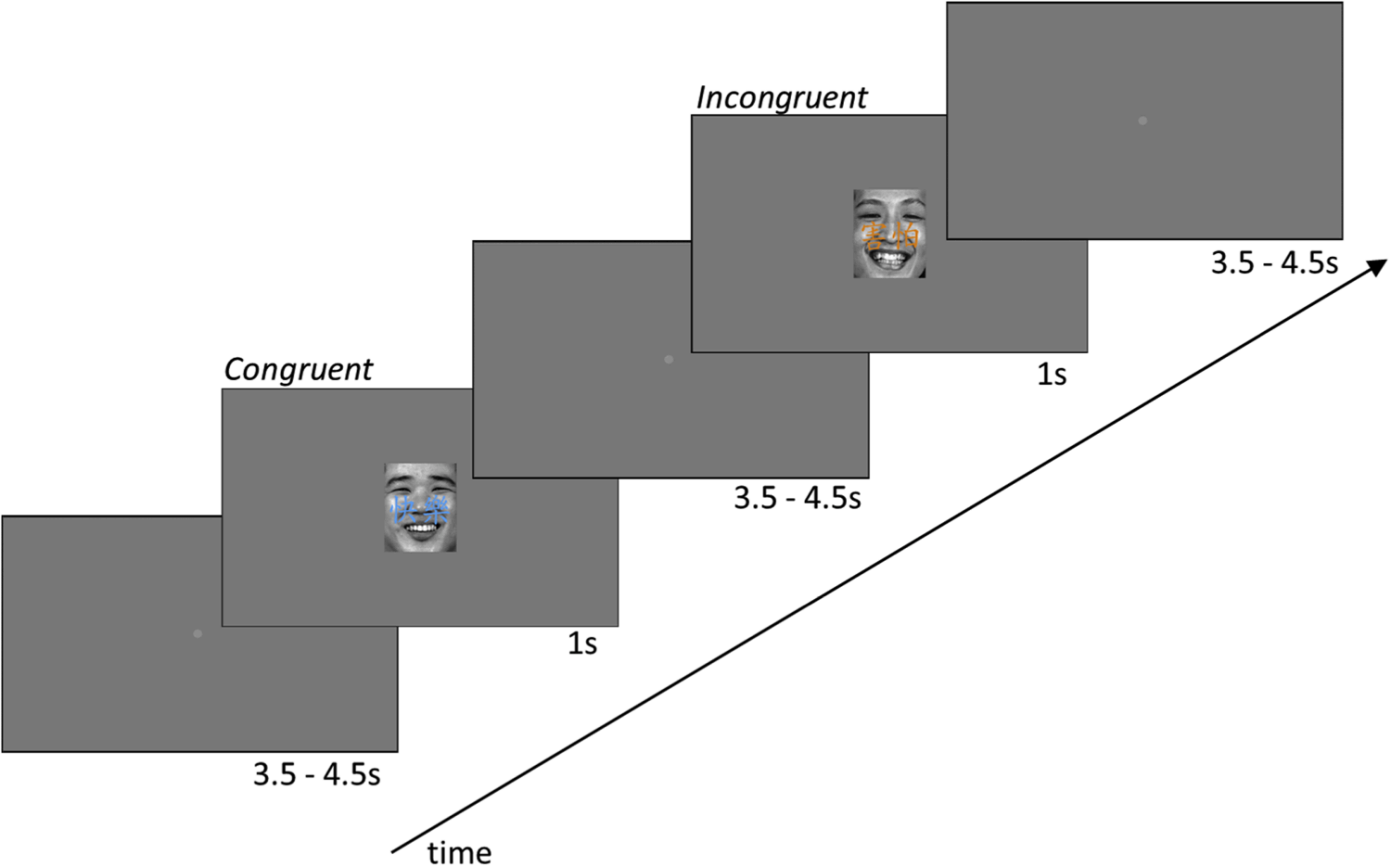
Experimental paradigm. Each trial began with a central fixation point on a gray background. After a delay, a face stimulus was presented with a superimposed colored emotional word for 1000 ms and participants were required to identify the facial expression while ignoring the overlaid word.

*Emotional face-word Stroop task.* Participants performed an emotional face–word Stroop task (Fig. 1) adapted from the paradigm described by Grueschow et al. (2021) and previously reported in detail (Chang et al., 2023). Each trial began with the presentation of a gray fixation point (FP; 0.5° diameter, 54 cd/m²) centered on a uniform gray background (6.5 cd/m²) for a variable duration of 4000–5500 ms. The FP then disappeared and was followed by an emotional facial stimulus (4.0° × 4.7°, 47 cd/m²; happy or fearful), overlaid with a colored Chinese emotion word (“happy” or “fearful”; 60 cd/m²; four possible colors), presented centrally for 1000 ms. Participants were instructed to categorize the facial expression (happy vs. fearful) while ignoring the superimposed word, which could be emotionally congruent (no-conflict trials) or incongruent (conflict trials) with the facial expression. Responses were made via keyboard using the “F” and “J” keys, mapped to happy and fearful responses, respectively; response–emotion mappings were counterbalanced across participants. The task comprised 240 trials, divided into two blocks of 120 trials. Facial identities, sex, and emotional expressions were randomized across trials, and stimulus occurrences were counterbalanced across trial types and response mappings.

*Stimuli.* Adult facial stimuli were selected from the Taiwan Facial Expression Image Database (Chen et al., 2013). The stimulus set comprised thirty front-facing adult models (10 male, 10 female), each displaying happy and fearful expressions. Consistent with previous studies (Soncin et al., 2016; Wang et al., 2018; Yep et al., 2018), all face images were converted to grayscale and spatially aligned to a central fixation point. To minimize potential confounding effects of low-level visual features on microsaccade responses, luminance, visual contrast, and spatial frequency were equated across stimuli using the SHINE MATLAB toolbox (Willenbockel et al., 2010). The mean luminance of the combined face–word stimuli was held constant across experimental conditions at approximately 65 cd/m².

**Fig. 2:**
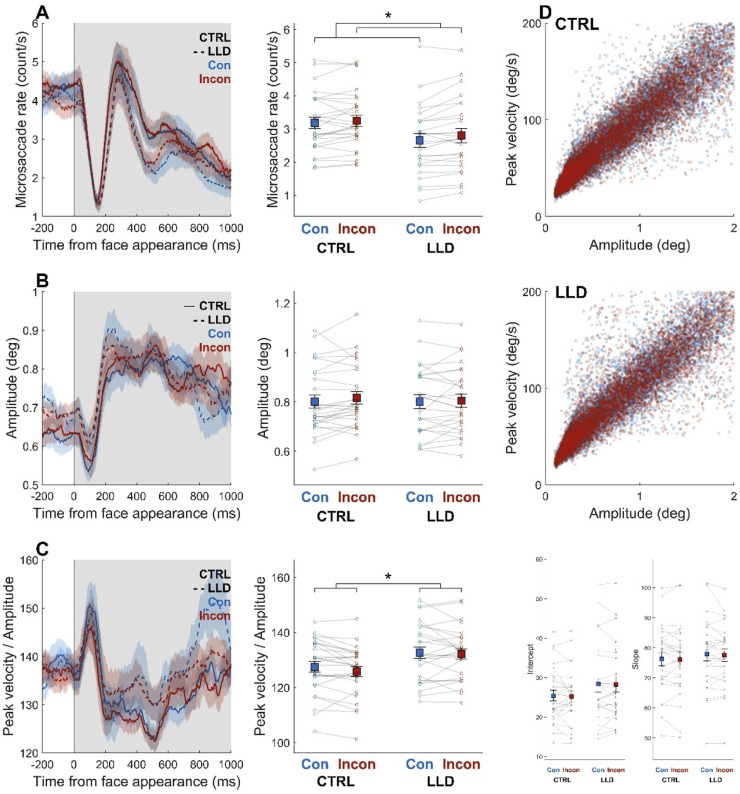
Time-resolved effects of emotional congruency on microsaccade kinematics. (A) Microsaccade occurrence dynamics following the face presentation and mean values of microsaccade rates in the face epoch (0 to 1000 ms) in different conditions and experimental groups. (B) Microsaccade amplitude dynamics and mean amplitude values in the face epoch (0 to 1000 ms) in different conditions and experimental groups. (C) Peak-velocity-to-amplitude-ratio dynamics and mean ratio values in the face epoch (0 to 1000 ms) in different conditions and experimental groups. (D) Microsaccade main sequence, intercept, and slope in different conditions and experimental groups. The shaded colored regions surrounding microsaccade dynamics curves represent the ± standard error range (across participants) for different conditions. The color-filled squares and error-bars represent mean value ± standard error (across participants) for each condition, and the small circles represent mean value for each subject. Color dots represent each data point. The gray area represents the epoch selected for analyses. Con: congruent condition. Incon: incongruent condition. CTRL: healthy older adults. LLD: late-life depression patients. * indicates differences are statistically significant.

*Data analysis.* Analyses focused on microsaccade behavior and included only correct-response trials. Microsaccades were identified using a well-established velocity-based detection algorithm (Engbert & Kliegl, 2003; Engbert & Mergenthaler, 2006), as implemented in our previous work (Barquero et al., 2023; Chen et al., 2021; Hsu et al., 2021; Wang et al., 2020; Wang & Munoz, 2021). Briefly, horizontal and vertical eye-position signals were used to compute eye velocity and movement duration. Velocity thresholds were determined adaptively for each trial based on noise estimates calculated from a ± 5000 ms window surrounding face onset, with microsaccades defined as events exceeding 6 median standard deviations of the velocity distribution. To reduce noise and ensure binocular validity, only microsaccades detected concurrently in both eyes for at least one sample were included. To exclude larger saccades, an amplitude criterion was applied, restricting analyses to movements with amplitudes between 0.1° and 2° Participants were required to have at least five trials (microsaccades) in each condition to be included in the analysis. As illustrated in the figures (e.g., Fig. 2D), the detected microsaccades exhibited the characteristic relationship between peak velocity and amplitude (i.e., the main sequence). Microsaccade amplitude (degrees), peak velocity (deg/s), and main-sequence parameters (slope and intercept of the peak velocity–amplitude relationship) were quantified. Following previous research (Buonocore et al., 2017), the peak-velocity-to-amplitude ratio was also calculated to characterize transient stimulus-evoked changes in microsaccade kinematics. The ratio was expressed in s⁻¹ and was treated as distinct from the regression-based main-sequence slope. Microsaccade rate was first computed at the individual-participant level by averaging across trials within each experimental condition, and rates were then averaged across participants. Consistent with previous (e.g., Engbert & Kliegl, 2003; Laubrock et al., 2005; Valsecchi & Turatto, 2009), microsaccade occurrences were converted into a rate-per-second measure using a sliding window of 100 ms. Temporal dynamics of microsaccade metrics were additionally visualized using a broader moving window of 100 ms; these smoothed time courses were used solely for illustrative purposes. Consistent with previous research (Chang et al., 2025), the epoch for emotional conflict or valence was set to 0 – 1000 ms after face appearance (referred to as the face epoch) to approximately capture the modulation of microsaccade rate and metrics by emotional processing. This epoch was used because the emotional stimulus was presented during this time. Based on our hypotheses that emotional conflict and valence would modulate microsaccade behavior and that these modulations might differ between LLD and CTRL participants, we conducted simple main-effect analyses within each group, where appropriate, in addition to testing the main and interaction effects. Effect sizes are reported where appropriate. Statistical analyses were conducted using R (R Core Team, 2020; Rstudio Team, 2019), JASP (JASP Team, 2019), and MATLAB (The MathWorks Inc., Natick, MA, USA).

The primary hypothesis-driven analyses were informed by our previous study using the same emotional face–word Stroop paradigm (Chang et al., 2025) and examined emotional congruency and valence effects on conventional microsaccade measures during the primary face-locked epoch (0–1000 ms) and pre-response epoch (−1000–0 ms). Omnibus tests for these primary analyses were evaluated using a two-sided significance threshold of p < .05, and Bonferroni-adjusted p values were used for within-group follow-up comparisons. Analyses of the peak-velocity-to-amplitude ratio and main-sequence parameters, finer phase- and interval-specific windows, the 350–1000-ms post-response period, inter-individual correlations, and classification performance were considered secondary. To limit inflation of Type I error, Benjamini–Hochberg false-discovery-rate correction was applied within conceptually defined families of secondary inferential tests, separately for emotional congruency and valence. These families comprised full-epoch kinematic extensions, phase-specific rate analyses, phase-specific main-sequence analyses, interval-specific kinematic analyses, post-response kinematic analyses, and correlations within each clinical or cognitive measure. Raw p values and FDR-adjusted q values are reported for these exploratory analyses. Findings with p < .05 but q ≥ .05 were considered nominal and hypothesis-generating. A single correction was not applied across all tests because the analysis families addressed distinct questions and included correlated measures.

To examine whether the principal microsaccade findings were robust to recorded medication exposure, we conducted secondary hierarchical linear mixed-effects sensitivity analyses (Braund et al., 2021). These analyses examined face-locked microsaccade rate for the emotional congruency and valence comparisons, pre-response microsaccade amplitude for the congruency comparison, and the face-locked peak-velocity-to-amplitude ratio for the congruency comparison. Each model included a participant-specific random intercept and binary fixed-effect indicators for medication classes with at least one exposed participant: SSRI, SNRI, NDRI, TCA, α-blocker, β-blocker, and benzodiazepine. Medication indicators were coded as 1 for current use and 0 for no recorded use. Beginning with a medication-covariate model, nested models sequentially added group, task condition, and the group × condition interaction and were compared using likelihood-ratio tests. Detailed model comparisons and fixed-effect estimates are reported in Supplementary Table 1. Given the modest sample size, uneven medication-class frequencies, and overlap between medication use and diagnostic group, these analyses were treated as sensitivity analyses rather than as tests of the effects of individual medication classes.

To explore whether eye-tracking indices derived from the emotional face–word Stroop paradigm could differentiate LLD from CTRL, we performed a supervised classification analysis using subject-level summary features computed from correct-response trials. This secondary analysis was exploratory and hypothesis-generating and was not designed to establish diagnostic utility. We constructed contrast indices capturing emotional congruency (incongruent–congruent; “incon–con”) and emotional valence ("fear–happy") for microsaccade rate, amplitude, peak velocity, and reaction time (RT). We also included pupil responses component scores according to principle component analysis (Com_1 and Com_2) reflecting low-dimensional autonomic response structure previously applied in LLD work to summarize condition effects, as well as eye blink rate contrast indices (incon–con; fear–happy) (Lee, Chang et al., 2025).

Classification followed the general strategy for eye-tracking–based clinical differentiation (Brien et al., 2023), in which multiple classifiers produce individual-level probabilities that can be combined via a voting/averaging rule, and performance is quantified using ROC-AUC. We implemented a nested cross-validation framework in MATLAB. In the outer loop (10-fold stratified CV), models were trained on 9 folds and tested on the held-out fold to generate out-of-fold predicted probabilities for every participant. Within each outer-training set, features were z-scored using training-set means/standard deviations (with missing values imputed by training-set means) and the same transformation was applied to the held-out test fold. Hyperparameters were selected in an inner CV loop (3-fold) by maximizing inner-CV AUC for three base learners: (a) RBF-kernel support vector machine (SVM), (b) ridge-regularized logistic regression, and (c) random forest (TreeBagger). In each outer fold, the three trained models produced held-out probabilities of LLD that were combined by soft voting (mean probability) to yield an “ensemble probability” per participant. ROC curves and AUC were computed from the pooled out-of-fold probabilities. A decision threshold was selected using Youden’s J statistic (maximizing sensitivity + specificity − 1) on the out-of-fold ROC to summarize accuracy, sensitivity, and specificity. To test whether observed ROC-AUC exceeded chance under small-sample conditions, we conducted a permutation test (1000 iterations) in which group labels were randomly shuffled and the outer-CV procedure was repeated to generate a null distribution of AUC values. In addition, to reduce model variance and assess robustness to model complexity, we repeated the outer-CV analysis using a single ridge-regularized logistic regression model (fixed regularization) and computed ROC-AUC from its out-of-fold probabilities.

## Results

### Modulation of microsaccade rate and metrics by emotional congruency

To examine whether microsaccade dynamics following face onset in the emotional face–word Stroop task were modulated by emotional conflict, and whether such modulation differed between groups, we analyzed microsaccade rate and kinematic metrics time-locked to face appearance using only trials with correct responses. In both groups, microsaccade rate exhibited the characteristic transient suppression following face-word onset, followed by a rebound increase later in the stimulus period (Fig. 2A). To quantify overall rate modulation, microsaccade rates during the face epoch (0–1000 ms post–face onset) were analyzed. Mean microsaccade rates were 3.19 ± 0.88 and 3.25 ± 0.87 counts/s for CTRL participants, and 2.65 ± 1.03 and 2.80 ± 1.09 counts/s for LLD participants, in the congruent and incongruent conditions, respectively. Across groups, microsaccade rates were significantly higher in the incongruent than in the congruent condition (F(1,51) = 9.96, p = 0.003, ηp² = 0.16). Microsaccade rates also tended to be lower in LLD than in CTRL participants, although this group effect did not reach statistical significance (F(1,51) = 3.47, p = 0.07, ηp² = 0.06). No significant group × congruency interaction was observed (F(1,51) = 1.62, p = 0.21, ηp² = 0.03). To further explore emotional conflict effects within each group, simple main effect analyses revealed that congruency effects were not significant in CTRL participants (p = 0.23), but were significantin the LLD group (p = 0.002).

As shown in Fig. 2B, microsaccade amplitude increased following face onset but showed no significant effects of group, congruency, or the group × congruency interaction (all p ≥ 0.16). To capture transient changes in microsaccadic velocity–amplitude scaling that may not be reflected by amplitude alone, we additionally examined the peak-velocity-to-amplitude ratio (Buonocore et al., 2017; Malevich et al., 2021). In this secondary analysis, ratios were nominally higher in LLD than in CTRL participants, F(1, 51) = 4.76, p = 0.034, q = 0.306, ηp² = 0.09, but this effect did not survive FDR correction. Neither the congruency effect nor the group × congruency interaction was significant (both q ≥ 0.329; Fig. 2C). Global main-sequence intercepts and slopes likewise showed no significant effects of group, congruency, or their interaction (all p ≥ 0.20; Fig. 2D).

To test whether full-epoch averages masked temporally localized effects, we conducted secondary phase- and interval-specific analyses. Microsaccade rate was quantified during inhibition (100–200 ms), rebound (200–400 ms), and post-rebound (400–600 ms) periods (Supplementary Fig. 1). Rates were comparable during inhibition and rebound, but were nominally lower in LLD than in CTRL participants during the post-rebound period, F(1, 51) = 4.81, p = 0.033, q = 0.297, ηp² = 0.09. This effect did not survive FDR correction, and no phase-specific main-sequence effect survived correction. We also examined amplitude, peak velocity, and the peak-velocity-to-amplitude ratio during early (0–200 ms), intermediate (200–500 ms), and later (500–1000 ms) intervals (Supplementary Fig. 2). No amplitude or peak-velocity effect survived correction. During the later interval, the ratio was nominally higher in LLD than in CTRL participants, F(1, 51) = 5.22, p = 0.026, q = 0.621, ηp² = 0.09, and lower in incongruent than congruent trials, F(1, 51) = 4.08, p = 0.049, q = 0.621, ηp² = 0.07; neither effect survived correction, and the interaction was nonsignificant. The present study focuses on microsaccade dynamics; corresponding effects of emotional conflict on behavioral performance, pupil responses, and eye-blink measures are reported in the Supplementary Information (Supplementary Fig. 3) and were used only for exploratory classification analysis.

### Emotional congruency modulation of microsaccade rate and metrics relative to the manual response

**Fig. 3:**
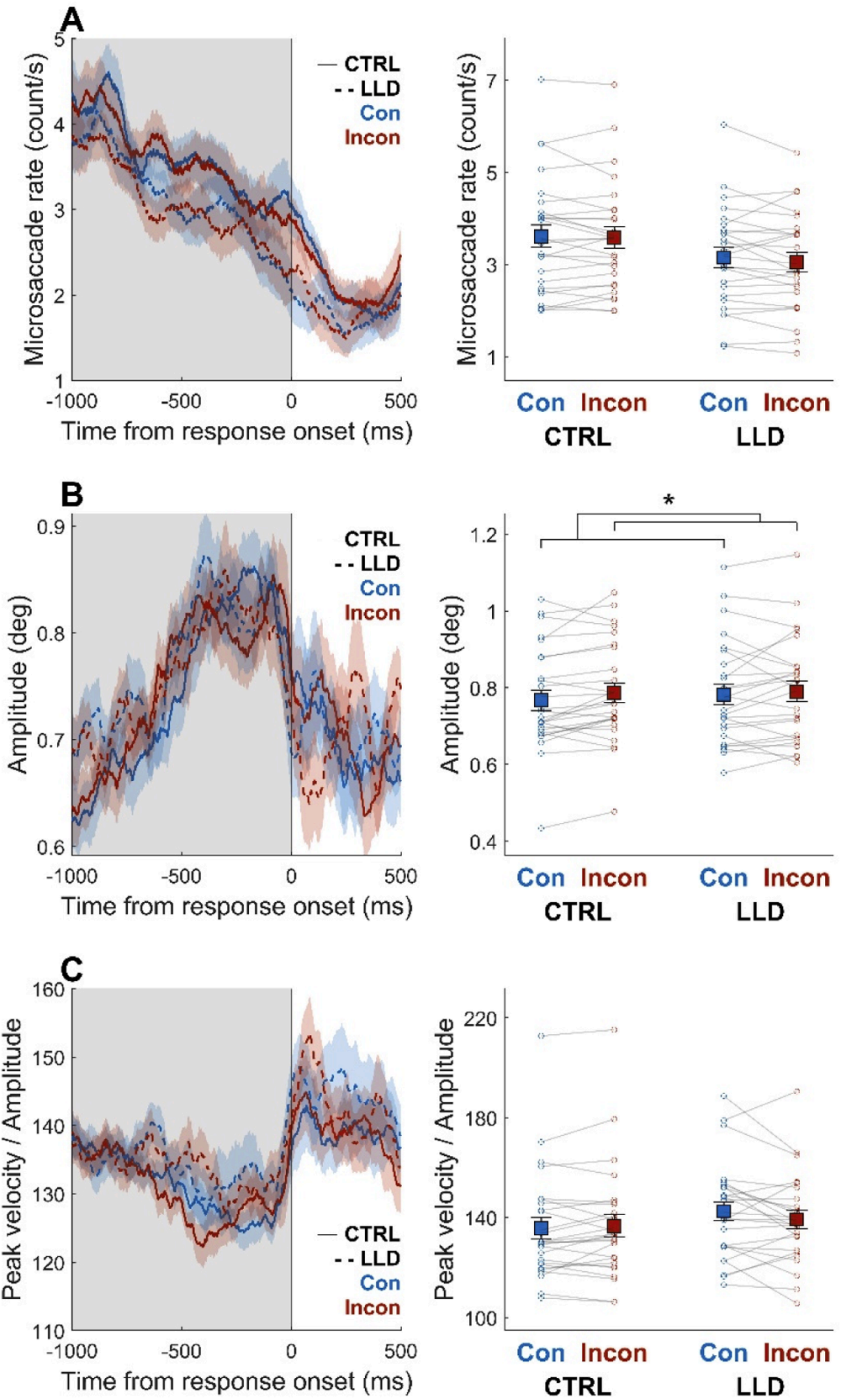
Emotional congruency modulation on microsaccade behavior before the manual response. (A) Microsaccade occurrence dynamics following response onset and mean values of microsaccade rates in the pre-response epoch (−1000 to 0 ms) in different conditions and experimental groups. (B) Microsaccade amplitude dynamics and mean amplitude values in the pre-response epoch (−1000 to 0 ms) in different conditions and experimental groups. (C) Peak-velocity-to-amplitude-ratio dynamics and mean ratio values in the pre-response epoch (−1000 to 0 ms) in different conditions and experimental groups. The shaded colored regions surrounding microsaccade dynamics curves represent the ± standard error range (across participants) for different conditions. The color-filled squares and error-bars represent mean value ± standard error (across participants) for each condition. The gray area represents the epoch selected for analyses. Con: congruent condition. Incon: incongruent condition. CTRL: healthy older adults. LLD: late-life depression patients. * indicates differences are statistically significant.

To further examine emotional-congruency effects during response preparation, we analyzed microsaccade rate and kinematic measures during the pre-response epoch (−1000 to 0 ms relative to response onset). Microsaccade rates decreased as participants approached the manual response (Fig. 3A). Mean rates were 3.61 ± 1.25 and 3.58 ± 1.19 counts/s in CTRL participants and 3.15 ± 1.09 and 3.06 ± 1.06 counts/s in LLD participants for the congruent and incongruent conditions, respectively. No significant effects of group, congruency, or their interaction were observed (all p ≥ 0.12). Microsaccade amplitude increased before response onset (Fig. 3B), with mean amplitudes of 0.77 ± 0.13 and 0.79 ± 0.13° in CTRL participants and 0.78 ± 0.14 and 0.79 ± 0.14° in LLD participants. Amplitude was significantly larger during incongruent than congruent trials, F(1, 51) = 4.37, p = 0.040, ηp² = 0.08, whereas the group effect and group × congruency interaction were nonsignificant (both p ≥ 0.36). Prespecified within-group analyses detected the congruency effect in CTRL participants (p = 0.020) but not in LLD participants (p = 0.480). As a secondary measure of velocity–amplitude scaling, we examined the peak-velocity-to-amplitude ratio, which decreased toward response onset (Fig. 3C). Mean ratios were 128.76 ± 9.93 and 127.09 ± 9.43 s⁻¹ in CTRL participants and 131.73 ± 11.51 and 131.23 ± 11.77 s⁻¹ in LLD participants. No effects of group, congruency, or their interaction survived FDR correction (all q ≥ 0.168). Finally, prompted by the apparent post-response amplitude increase in LLD, we examined amplitude and the ratio from 350 to 1000 ms after response onset. Amplitude was numerically higher in LLD than in CTRL participants, but the group effect did not survive correction, F(1, 51) = 2.99, p = 0.090, q = 0.260, ηp² = 0.06. No other post-response effect survived FDR correction (all q ≥ 0.260; Supplementary Fig. 4).

### Modulation of microsaccade rate and metrics by emotional valence

**Fig. 4:**
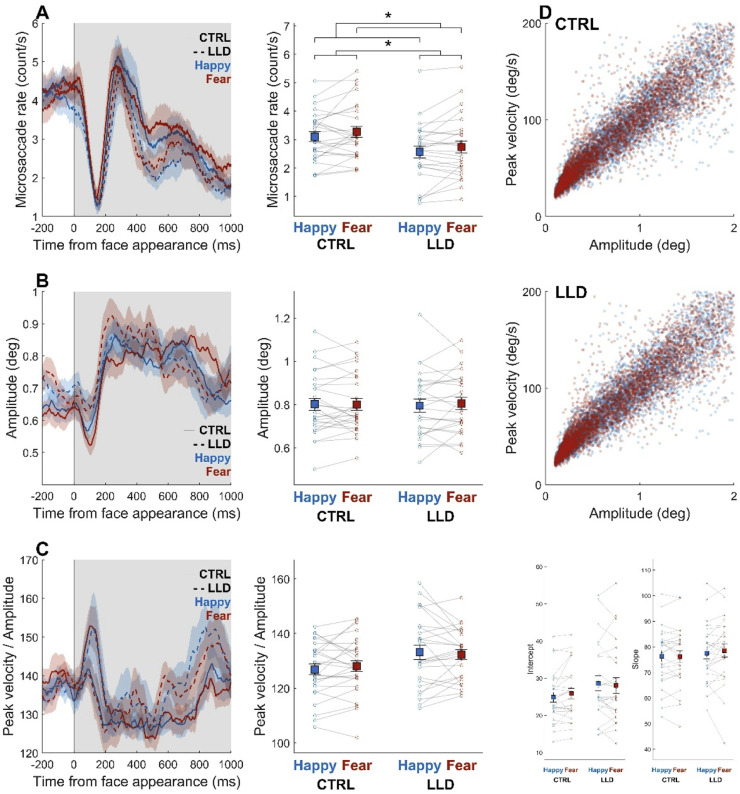
Effect of emotional valence on microsaccade behavior. (A) Microsaccade occurrence dynamics following the face presentation and mean values of microsaccade rates in the face epoch (0 to 1000 ms) in different conditions and experimental groups. (B) Microsaccade amplitude dynamics and mean amplitude values in the face epoch (0 to 1000 ms) in different conditions and experimental groups. (C) Peak-velocity-to-amplitude-ratio dynamics and mean ratio values in the face epoch (0 to 1000 ms) in different conditions and experimental groups. (D) Microsaccade main sequence, intercept, and slope in different conditions and experimental groups. The shaded colored regions surrounding microsaccade dynamics curves represent the ± standard error range (across participants) for different conditions. The color-filled squares and error-bars represent mean value ± standard error (across participants) for each condition, and the small circles represent mean value for each subject. Color dots represent each data point. The gray area represents the epoch selected for analyses. Happy: happy condition. Fear: fear condition. CTRL: healthy older adults. LLD: late-life depression patients. * indicates differences are statistically significant.

To examine emotional-valence effects following face onset, we analyzed microsaccade rate and kinematic measures during the face epoch using correct congruent trials. Mean rates were 3.10 ± 0.86 and 3.28 ± 0.97 counts/s in CTRL participants and 2.57 ± 1.03 and 2.74 ± 1.09 counts/s in LLD participants for the happy and fear conditions, respectively (Fig. 4A). Rates were significantly higher in the fear than in the happy condition, F(1, 51) = 6.57, p = 0.013, ηp² = 0.11, and significantly lower in LLD than in CTRL participants, F(1, 51) = 4.14, p = 0.047, ηp² = 0.08. The group × valence interaction was nonsignificant, F(1, 51) = 0.00, p = 0.990, ηp² = 0.00. Prespecified within-group analyses indicated that the valence effect approached significance in LLD participants (p = 0.058) but was nonsignificant in CTRL participants (p = 0.100).

Microsaccade amplitude increased following face onset (Fig. 4B), with mean amplitudes of 0.80 ± 0.14 and 0.80 ± 0.14° in CTRL participants and 0.79 ± 0.15 and 0.81 ± 0.14° in LLD participants. No effects of group, valence, or their interaction were observed (all p ≥ 0.58). As a secondary measure of stimulus-evoked velocity–amplitude scaling, we examined the peak-velocity-to-amplitude ratio (Fig. 4C). Mean ratios were 126.89 ± 10.12 and 128.08 ± 10.40 s⁻¹ in CTRL participants and 133.16 ± 13.08 and 132.24 ± 9.22 s⁻¹ in LLD participants. No effect survived FDR correction. The group effect did not reach significance, F(1, 51) = 3.46, p = 0.069, q = 0.311, ηp² = 0.06, and the valence and interaction effects were also nonsignificant (both p ≥ 0.279). Main-sequence intercepts and slopes showed no significant effects (all p ≥ 0.060; Fig. 4D). Because the intercept interaction approached significance, follow-up analyses were conducted; valence modulation approached significance in CTRL participants (p = 0.054) but was nonsignificant in LLD participants (p = 0.400).

To test whether full-epoch averages masked temporally localized effects, we conducted secondary phase- and interval-specific analyses. Rates were comparable between groups during the inhibition (100–200 ms) and rebound (200–400 ms) periods, but were nominally lower in LLD during the post-rebound period (400–600 ms), F(1, 51) = 5.80, p = 0.020, q = 0.180, ηp² = 0.10 (Supplementary Fig. 5). A nominally higher rebound-period main-sequence intercept was also observed in LLD, F(1, 51) = 4.22, p = 0.045, q = 0.516, ηp² = 0.08. Neither effect survived FDR correction, and no other phase-specific effect survived correction. Interval-specific analyses of the early (0–200 ms), intermediate (200–500 ms), and later (500–1000 ms) periods identified nominal group × valence interactions during the intermediate interval for amplitude, F(1, 51) = 5.98, p = 0.018, q = 0.198, ηp² = 0.11, and peak velocity, p = 0.022, q = 0.198 (Supplementary Fig. 6). During the later interval, the ratio was nominally higher in LLD than in CTRL participants, F(1, 51) = 6.40, p = 0.015, q = 0.198, ηp² = 0.11. None of these interval-specific effects survived FDR correction.

### Emotional valence modulation of microsaccade rate and metrics relative to the manual response

**Fig. 5:**
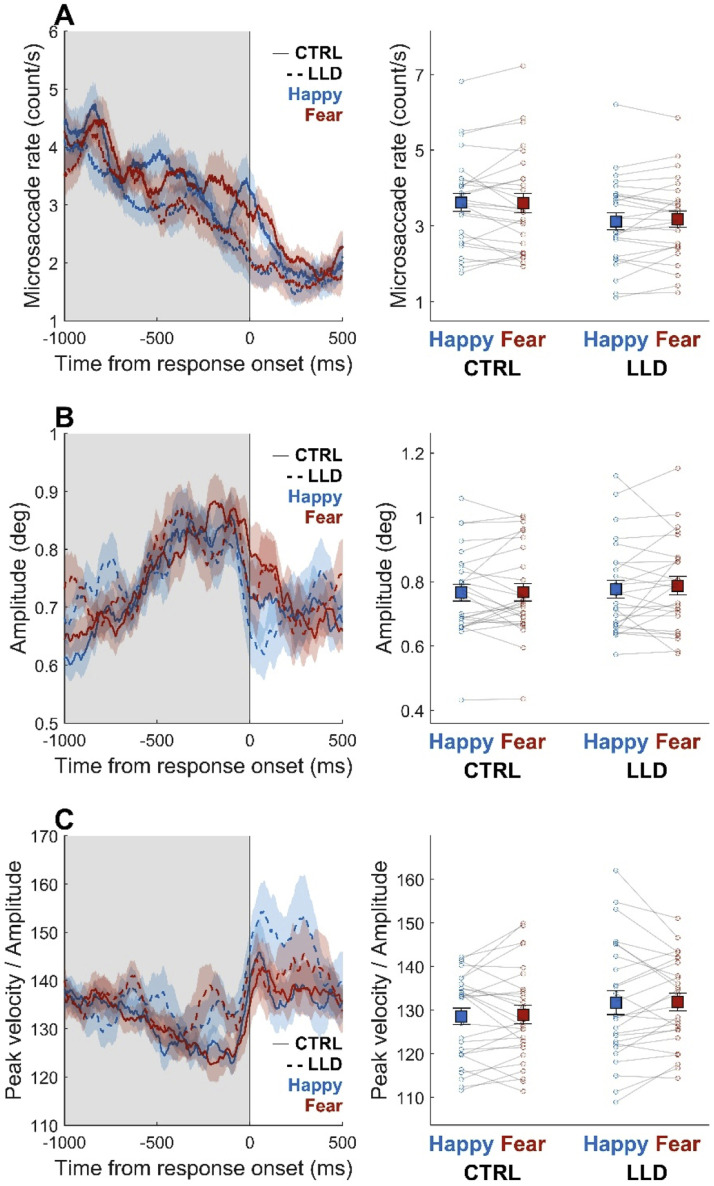
Emotional valence modulation on microsaccade behavior before the manual response. (A) Microsaccade occurrence dynamics following response onset and mean values of microsaccade rates in the pre-response epoch (−1000 to 0 ms) in different conditions and experimental groups. (B) Microsaccade amplitude dynamics and mean amplitude values in the pre-response epoch (−1000 to 0 ms) in different conditions and experimental groups. (C) Peak-velocity-to-amplitude-ratio dynamics and mean ratio values in the pre-response epoch (−1000 to 0 ms) in different conditions and experimental groups. The shaded colored regions surrounding microsaccade dynamics curves represent the ± standard error range (across participants) for different conditions. The color-filled squares and error-bars represent mean value ± standard error (across participants) for each condition. The gray area represents the epoch selected for analyses. Happy: happy condition. Fear: fear condition. CTRL: healthy older adults. LLD: late-life depression patients. * indicates differences are statistically significant.

To examine emotional-valence effects during response preparation, we analyzed microsaccade rate and kinematic measures during the pre-response epoch. Microsaccade rates decreased as participants approached the manual response (Fig. 5A). Mean rates were 3.62 ± 1.21 and 3.60 ± 1.34 counts/s in CTRL participants and 3.12 ± 1.14 and 3.18 ± 1.08 counts/s in LLD participants for the happy and fear conditions, respectively. No significant effects of group, valence, or their interaction were observed (all p ≥ 0.16). Microsaccade amplitude increased before response onset (Fig. 5B), with mean amplitudes of 0.77 ± 0.13 and 0.77 ± 0.14° in CTRL participants and 0.78 ± 0.14 and 0.79 ± 0.14° in LLD participants. Amplitude showed no effects of group, valence, or their interaction (all p ≥ 0.55). As a secondary measure of velocity–amplitude scaling, we examined the peak-velocity-to-amplitude ratio, which decreased toward response onset (Fig. 5C). Mean ratios were 128.56 ± 9.83 and 128.96 ± 10.83 s⁻¹ in CTRL participants and 131.73 ± 13.64 and 131.84 ± 10.03 s⁻¹ in LLD participants. No effects of group, valence, or their interaction were observed, and none survived FDR correction (all q = 0.860). In a secondary analysis of the 350–1000-ms post-response interval, amplitude showed a nominal valence effect, with larger amplitudes in the fear than in the happy condition, F(1, 51) = 4.61, p = 0.037, q = 0.126, ηp² = 0.08. The group effect, p = 0.063, q = 0.126, and group × valence interaction, p = 0.058, q = 0.126, also did not survive correction. No effect on the peak-velocity-to-amplitude ratio survived FDR correction (all q > 0.33; Supplementary Fig. 7).

### Inter-individual correlations between task-evoked microsaccade behavior and neuropsychological measures

**Fig. 6:**
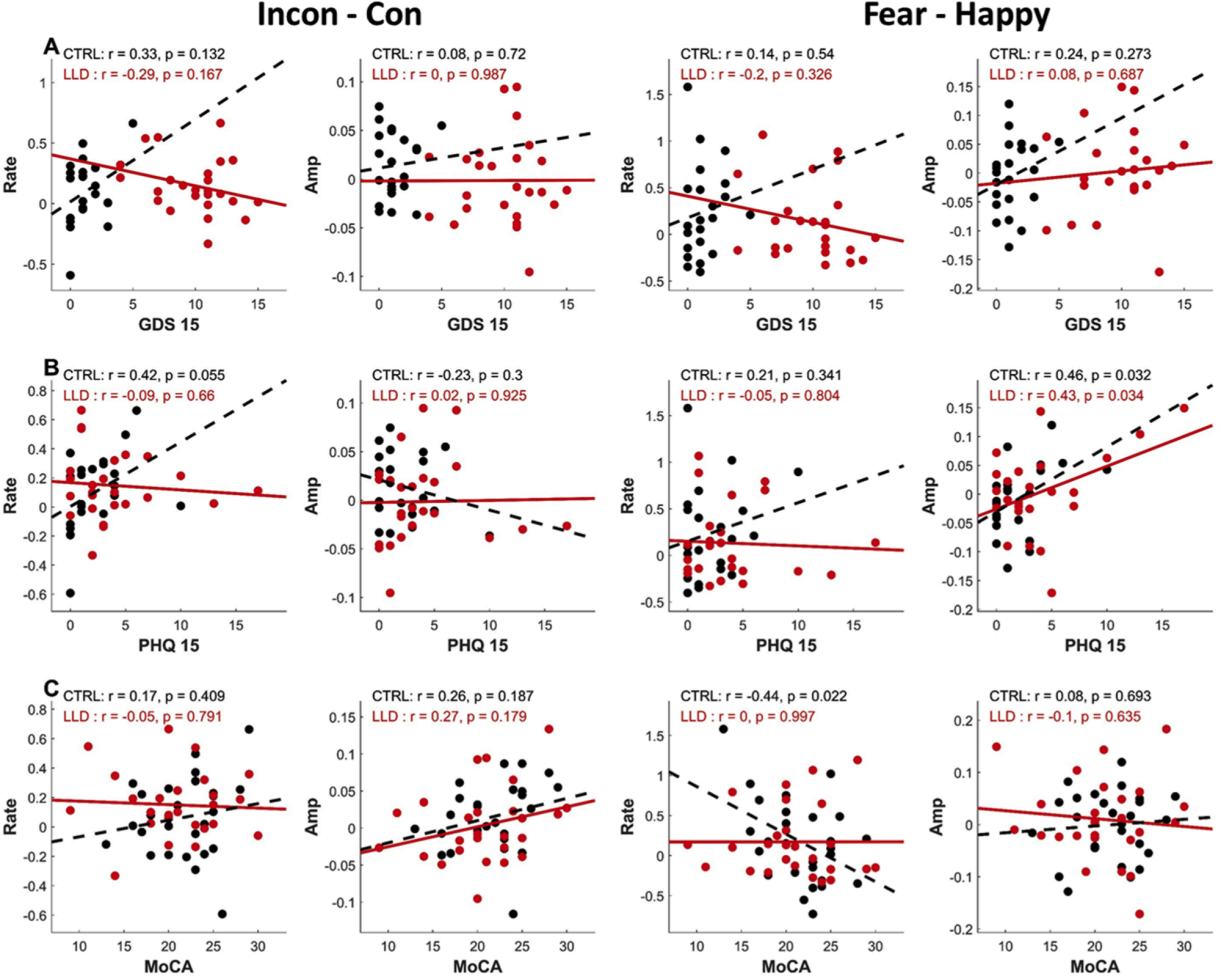
Inter-individual correlations on emotional congruency and valence effects between task-evoked microsaccade behavior and neuropsychological test scores. (A) Inter-individual correlation on emotional congruency (incongruent minus congruent condition) and valence (fear minus happy condition) effects between GDS-15 scores and microsaccade rate and amplitude. (B) Inter-individual correlation on emotional congruency and valence effects between PHQ-15 scores and microsaccade rate and amplitude. (C) Inter-individual correlation on emotional congruency and valence effects between MoCA scores and microsaccade rate and amplitude. The small circles represent mean value for each subject. Lines indicate the regression lines. Incon - Con: incongruent condition minus congruent condition. Fear - Happy: fear condition minus happy condition. GDS: Geriatric Depression Scale. PHQ: Patient Health Questionnaire. MoCA: Montreal Cognitive Assessment. Amp: amplitude. r: Pearson correlation coefficient. p: p value. CTRL: healthy older adults. LLD: late-life depression patients.

To explore the clinical relevance of task-evoked microsaccade modulation, we examined associations with depressive symptoms, somatic symptom severity, and global cognitive status. These exploratory analyses focused on theoretically motivated congruency (incongruent–congruent) and valence (fear–happy) contrasts in microsaccade rate and amplitude, examined separately within the CTRL and LLD groups with FDR correction applied within each neuropsychological measure. No association with GDS-15 scores survived correction (all q > 0.65; Fig. 6A). For PHQ-15 scores, congruency-related rate modulation showed a positive trend in CTRL participants, r = 0.42, p = 0.055, q = 0.147. Valence-related amplitude modulation was nominally positively associated with PHQ-15 scores in both CTRL participants, r = 0.46, p = 0.032, q = 0.136, and LLD participants, r = 0.43, p = 0.034, q = 0.136 (Fig. 6B). For MoCA scores, valence-related rate modulation showed a nominal negative association in CTRL participants, r = −0.44, p = 0.022, q = 0.176, but not in LLD participants, r = 0.00, p = 0.990 (Fig. 6C). No other association reached nominal significance. Overall, none survived FDR correction, and these patterns should therefore be considered exploratory and hypothesis-generating.

### Medication-adjusted sensitivity analyses

Hierarchical mixed-effects sensitivity analyses indicated that the principal task effects were retained after adjustment for recorded medication classes (Supplementary Table 2). For face-locked microsaccade rate in the congruency analysis, adding group, χ²(1) = 4.70, p = 0.030, and congruency, χ²(1) = 9.06, p = 0.003, improved model fit, whereas the group × congruency interaction did not, p = 0.199. In the resulting model, the congruency effect remained significant (p = 0.003), and the adjusted group coefficient reached significance (p = 0.049). Similarly, for face-locked rate in the valence analysis, adding group, χ²(1) = 5.80, p = 0.016, and valence, χ²(1) = 6.43, p = 0.011, improved model fit, whereas the interaction did not (p = 0.994). Both the adjusted group and valence effects remained significant (p = 0.029 and 0.013, respectively). The pre-response congruency effect on microsaccade amplitude was also retained after medication adjustment, χ²(1) = 4.36, p = 0.037; fixed-effect p = 0.040. For the secondary face-locked peak-velocity-to-amplitude ratio, adding group improved model fit, χ²(1) = 4.04, p = 0.044, but the adjusted group coefficient remained nonsignificant (p = 0.069). No individual medication-class coefficient was significant. Thus, the principal findings were not eliminated by adjustment for recorded medication classes, although the uneven medication exposures limit conclusions regarding medication-specific effects.

### Exploratory classification analysis of late-life depression

**Fig. 7:**
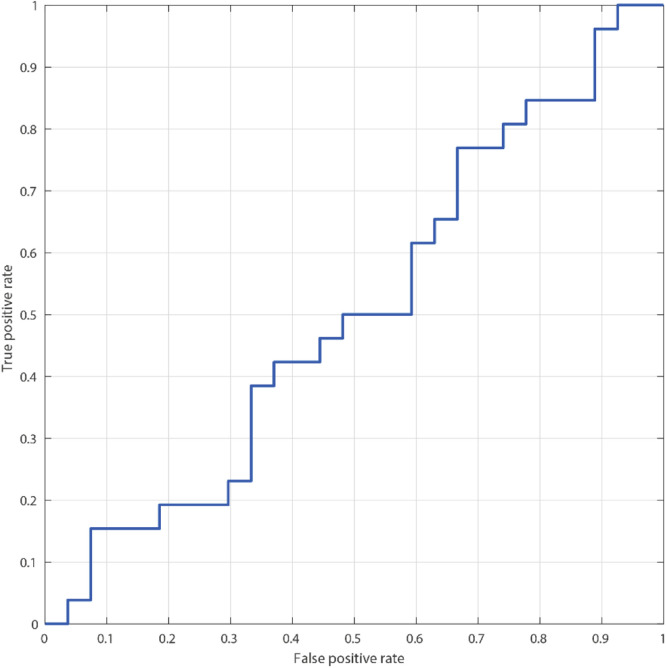
Receiver operating characteristic curve for the exploratory classification analysis differentiating late-life depression from healthy controls. The curve is based on out-of-fold predicted probabilities obtained from nested 10-fold cross-validation. The dashed diagonal indicates chance-level performance. AUC = 0.504; thr* indicates the Youden-optimized decision threshold (0.488).

To assess whether eye-tracking measures could differentiate individuals with LLD from CTRL participants, we performed an exploratory ROC/ML analysis using theory-driven congruency and valence contrasts in reaction time, microsaccade rate, amplitude, and peak velocity, together with pupil-response components and eye-blink indices previously examined in LLD (Y.T. Lee, Chang et al., 2025). Under nested 10-fold cross-validation with out-of-fold predictions, the three-model soft-voting ensemble performed at chance level (ROC–AUC = 0.504; Fig. 7). At the Youden-optimized threshold of 0.488, accuracy was 0.547, sensitivity was 0.769, and specificity was 0.333, indicating a tendency to classify participants as LLD (Table 2). Fold-wise accuracy was also highly variable (mean = 0.493, SD = 0.229). Permutation testing confirmed that performance did not differ from chance (p = 0.383), and a ridge-regularized logistic-regression sensitivity analysis yielded a comparable AUC of 0.500. In exploratory univariate analyses, the highest AUC was observed for the incongruent–congruent reaction-time contrast (AUC = 0.648), followed by the corresponding microsaccade-rate (AUC = 0.595) and eye-blink-rate contrasts (AUC = 0.593), whereas most remaining indices were near or below chance (AUC range = 0.35–0.52; Supplementary Table 3). Overall, the selected feature set did not support reliable individual-level classification of LLD and CTRL participants under out-of-sample validation.

**Table 2:** Summary classification metrics.

Metric	Value
ROC-AUC	0.504
Accuracy	0.547
Sensitivity	0.769
Specificity	0.333
Permutation p-value	0.383
Ridge logistic AUC	0.5

Note. Performance metrics are based on out-of-fold predictions from nested 10-fold cross-validation. Sensitivity and specificity correspond to the Youden-optimized decision threshold (0.488). The permutation p-value reflects the proportion of label-shuffled iterations (1000 permutations) yielding an AUC at least as large as the observed value.

## Discussion

In the present study, we examined whether task-evoked microsaccade dynamics, a behavioral oculomotor output generated through midbrain orienting circuitry, are altered in late-life depression during emotional conflict and valence processing in the emotional face–word Stroop task. At the group level, microsaccade rates were higher in the incongruent than in the congruent condition across participants and tended to be lower in individuals with LLD than in CTRL, although the group effect was not significant. Conventional microsaccade kinematic measures, including amplitude and main-sequence parameters, were largely comparable between groups. Secondary analyses of the peak-velocity-to-amplitude ratio and finer temporal intervals identified several nominal effects, but none survived FDR correction. During emotional valence processing, microsaccade rates were higher for fearful than happy faces and were significantly lower in LLD than in CTRL participants. At the inter-individual level, exploratory analyses identified nominal associations between selected microsaccade measures and somatic symptom burden or cognitive status, but none survived correction for multiple comparisons. Finally, an exploratory multivariate classification analysis combining microsaccade, pupil, eye-blink, and reaction-time indices yielded chance-level performance under cross-validated and permutation-tested conditions, indicating that the task-derived measures examined here did not support reliable individual-level classification. Together, these findings indicate that LLD is characterized by selective alterations in how emotional conflict and valence modulate microsaccade rate and metrics, highlighting microsaccades as an objective oculomotor index of emotional interference processing in ageing and depression.

### Altered microsaccade responses in late-life depression

Microsaccades have been linked to affective and conflict-related processing (e.g., Dalmaso et al., 2019; Krejtz et al., 2020), and recent work in healthy young adults has shown that their dynamics are systematically modulated during the emotional face–word Stroop task (Chang et al., 2025). Extending this work, the present findings indicate that microsaccade rate was sensitive to emotional conflict and valence, with lower overall rates in LLD during valence processing, whereas conventional kinematic measures were largely preserved. Following face onset, both groups exhibited the canonical pattern of rapid microsaccade suppression followed by a rebound increase. Rates were higher during incongruent than congruent trials across groups. Prespecified within-group analyses detected this congruency effect in LLD but not CTRL participants; however, because the group × congruency interaction was nonsignificant, this pattern does not establish stronger conflict-related modulation in LLD. Emotional conflict produced no significant group × congruency interactions in amplitude, peak velocity, or global main-sequence parameters. Although secondary analyses identified several nominal time-specific effects involving the peak-velocity-to-amplitude ratio, none survived FDR correction. Thus, during face processing, emotional conflict was expressed more consistently in microsaccade occurrence than in conventional kinematic measures.

During valence processing, microsaccade rates were higher for fearful than happy faces across groups and were significantly lower overall in LLD than in CTRL participants. This group difference is compatible with altered arousal–control regulation during emotional processing in depression, consistent with broader evidence of autonomic dysregulation such as reduced heart-rate variability (Gorman & Sloan, 2000). In healthy adults, lower microsaccade rates, particularly when accompanied by larger amplitudes or higher peak velocities, have been associated with heightened arousal or attentional engagement (Chen et al., 2021; Di Stasi et al., 2013, 2015; Siegenthaler et al., 2014). Accordingly, the lower rates observed in LLD may reflect heightened or less flexibly regulated engagement with emotional stimuli. During response preparation, rates decreased as the manual response approached, but neither congruency nor valence produced reliable between-group differences. Microsaccade amplitude was nevertheless larger during incongruent than congruent trials across groups. This effect was detected within CTRL but not LLD participants, which may suggest attenuation of preparatory kinematic modulation in LLD; however, the nonsignificant group × congruency interaction makes this interpretation tentative. This task-dependent pattern complements previous findings of larger microsaccade amplitudes and higher peak velocities in LLD during fixation under varying background luminance (Lee, Tai et al., 2025). Taken together, microsaccade rate and kinematics may reflect partially dissociable aspects of oculomotor control: rate effects were more consistently expressed during emotional stimulus processing, whereas kinematic effects appeared more context-dependent and may emerge under specific sensory or response-preparatory demands.

### Potential LC–NE and SC contributions to microsaccade modulation

Microsaccade generation depends on oculomotor circuitry involving the SC, a midbrain structure that integrates multisensory salience, cognitive-control, and arousal-related signals to guide orienting behavior (Basso & May 2017; Corneil & Munoz, 2014; Chin-An Wang & Munoz, 2024; White & Munoz, 2011). The SC is anatomically positioned to receive influences relevant to emotional conflict and valence processing through projections from the LC, ACC, and DLPFC (Edwards et al., 1979; Goldman & Nauta, 1976; Leichnetz et al., 1981; Li et al., 2018). Within this framework, two non-mutually exclusive mechanisms may contribute to the observed microsaccade patterns. First, altered LC–NE-related arousal regulation in LLD could influence gain signals affecting SC excitability and, consequently, the probability of microsaccade generation in response to emotionally salient stimuli. Because microsaccade rate is sensitive to arousal and attentional engagement (J.T. Chen et al., 2021; Dalmaso et al., 2019; Krejtz et al., 2020; Siegenthaler et al., 2014), this account is compatible with the lower overall rate observed in LLD during valence processing.

Second, altered top–down control involving the ACC and DLPFC could influence how emotional interference is translated into SC-related orienting output. Under this account, LLD may involve less flexible gating of microsaccade initiation or weaker expression of control demands in pre-response kinematic adjustments. The finding that congruency-related amplitude modulation was detected in CTRL but not LLD participants is compatible with this possibility, although the nonsignificant group × congruency interaction makes the interpretation tentative. Thus, the emotional face–word Stroop task provides a useful behavioral context for examining microsaccade responses that may be shaped by distributed arousal and control systems. Microsaccades offer a temporally precise behavioral readout of oculomotor output through which these influences on orienting behavior can be investigated; however, as indirect behavioral measures, they do not directly establish dysfunction within the LC–NE system, SC, ACC, or DLPFC.

### Exploratory inter-individual correlations with clinical and cognitive measures

Our exploratory correlational analyses examined whether task-evoked microsaccade modulation was associated with depressive symptom severity, somatic symptom burden, or global cognitive function. At the uncorrected level, selected microsaccade measures showed nominal associations with PHQ-15 and MoCA scores, whereas no comparable associations were observed for GDS-15 scores. However, none of these associations survived FDR correction within the corresponding neuropsychological-measure family. The present findings consequently do not establish that microsaccade dynamics preferentially track somatic or cognitive dimensions rather than depressive symptom severity. Although the nominal patterns are broadly compatible with previous LLD eye-tracking studies reporting associations with specific clinical or cognitive dimensions (Lee et al., 2024; Lee, Chang et al., 2025), they should be regarded as hypothesis-generating and require replication in larger, independent samples using prespecified measures and correlation hypotheses.

### Interpretation of the exploratory classification analysis

The exploratory ROC/ML analysis provided no evidence that the selected task-derived features could reliably distinguish LLD from CTRL at the individual level. The models were intentionally restricted to theory-driven contrast indices, such as incongruent–congruent and fear–happy differences, to emphasize task-evoked modulation rather than absolute condition-wise values. Despite this principled feature selection, convergent chance-level performance across the ensemble classifier, ridge-logistic sensitivity analysis, and permutation testing indicated that the current feature set, although sensitive to experimental manipulations (Lee, Chang et al., 2025), did not yield a sufficiently distinctive and reproducible multivariate signature for individual-level group classification. Although some individual indices showed numerically higher univariate AUCs, these analyses were exploratory, were not independently validated, and should not be interpreted as evidence of diagnostic utility. More broadly, the findings illustrate that statistically detectable condition- or group-level effects do not necessarily translate into stable individual-level classification. Given the modest sample size and absence of an independent validation cohort, the present analysis should be regarded as hypothesis-generating. Future studies may examine prespecified feature sets, including absolute condition-wise, longitudinal, or multimodal measures, in larger independent samples using preregistered models and external validation. At present, however, these results do not support the use of the oculomotor and autonomic measures derived from this emotional face–word Stroop task for diagnostic classification of LLD.

### Limitations and future directions

Several limitations should be acknowledged. First, although microsaccade generation depends on oculomotor circuitry that includes the SC, the present behavioral measures cannot isolate SC activity and should not be considered direct measures or validated proxies of LC–NE function. The proposed contributions of the LC–NE system, SC, ACC, and DLPFC therefore remain inferential. Future studies combining microsaccade measures with pharmacological manipulations, autonomic recordings, or neuroimaging will be needed to determine whether the observed behavioral differences are related to activity or connectivity within these neural systems. Second, medication use and residual clinical heterogeneity may have influenced microsaccade measures. Medication-adjusted mixed-effects sensitivity analyses indicated that the principal congruency and valence effects on microsaccade rate, as well as the pre-response congruency effect on amplitude, were retained after accounting for recorded medication classes. Nevertheless, medication exposure was nonrandom and substantially overlapped with diagnostic group. Variation in symptom profiles, treatment response, and medical comorbidity may also have contributed to the observed patterns. Larger, better-stratified samples will therefore be needed to distinguish illness-related from treatment-related effects. Finally, the number of oculomotor outcomes and statistical tests was relatively large in relation to the sample size. The primary congruency and valence analyses during the 0–1000-ms face epoch were hypothesis-driven and informed by our previous study using the same emotional face–word Stroop paradigm (Chang et al., 2025). In contrast, analyses of the peak-velocity-to-amplitude ratio, main-sequence parameters, finer phase- and interval-specific windows, the post-response period, inter-individual correlations, and classification performance were secondary or exploratory. To limit Type I error, Benjamini–Hochberg FDR correction was applied within conceptually related families of secondary tests, and Bonferroni adjustment was used for relevant pairwise follow-up comparisons. None of the secondary temporal, kinematic-extension, post-response, or correlational findings survived FDR correction; effects significant only at the uncorrected level were therefore treated as nominal and hypothesis-generating. Even so, family-based correction cannot fully eliminate the risk of Type I error, particularly given the modest sample size and correlations among the oculomotor measures. Future studies should preregister a restricted set of primary outcomes and temporal windows and evaluate them in larger, independent samples.

### Conclusion

In summary, this study shows that microsaccades in the emotional face–word Stroop task carry signatures of emotional conflict and valence processing in older adults, and that late-life depression is associated with selective alterations in these signatures. By examining an oculomotor output generated through circuitry that includes the SC and may be influenced by arousal and cortical control processes, our findings extend prior work and highlight microsaccades as a temporally precise, yet underexplored, behavioral measure of affective interference control in clinical ageing. These findings are consistent with possible contributions from LC–NE and SC-related circuitry but do not provide direct evidence of dysfunction within these neural systems.

## Authors contribution

All authors contributed to the study conception; C.W. designed research; Y.W. and Y.L. performed research; Y.C. and C.W. analyzed data; C.W. wrote the first draft of the manuscript; all authors provided comments and edits on various drafts of the manuscript.

## Statement

During the preparation of this work, we used generative AI technologies to edit the English text.

## Declaration of competing interest

The authors report no conflicts of interest.

## Data Availability

Data are available from the authors upon reasonable request following the publication.

## Appendix Supplementary materials

Image, application 1

## References

[bib0001] Adriani B., Arena J.F., Fioretti A., Mancino S., Sarno F., Ferracuti S., Del Casale A. (2024). Current diagnostic challenges in late-life depression and neurocognitive disorders. Psychiatry International.

[bib0002] Alexopoulos G.S. (2019). Mechanisms and treatment of late-life depression. Translational Psychiatry.

[bib0003] Aston-Jones G., Cohen J.D. (2005). An integrative theory of locus coeruleus-norepinephrine function: Adaptive gain and optimal performance. Annual Review of Neuroscience.

[bib0004] Aziz R., Steffens D.C. (2013). What are the causes of late-life depression?. Psychiatric Clinics of North America.

[bib0005] Barquero C., Chen J.T., Munoz D.P., Wang C.A. (2023). Human microsaccade cueing modulation in visual- and memory-delay saccade tasks after theta burst transcranial magnetic stimulation over the frontal eye field. Neuropsychologia.

[bib0006] Basso M.A., May P.J. (2017). Circuits for action and cognition: A view from the superior colliculus. Annual Review of Vision Science.

[bib0007] Bernard R., Kerman I.A., Thompson R.C., Jones E.G., Bunney W.E., Barchas J.D., Schatzberg A.F., Myers R.M., Akil H., Watson S.J. (2011). Altered expression of glutamate signaling, growth factor, and glia genes in the locus coeruleus of patients with major depression. Molecular Psychiatry.

[bib0008] Braund T.A., Tillman G., Palmer D.M., Gordon E., Rush A.J., Harris A.W.F. (2021). Antidepressant side effects and their impact on treatment outcome in people with major depressive disorder: An iSPOT-D report. Translational Psychiatry.

[bib0009] Brien D.C., Riek H.C., Yep R., Huang J., Coe B., Areshenkoff C., Grimes D., Jog M., Lang A., Marras C. (2023). Classification and staging of Parkinson’s disease using video-based eye tracking. Parkinsonism & Related Disorders.

[bib0010] Buonocore A., Chen C.Y., Tian X., Idrees S., Münch T.A., Hafed Z.M. (2017). Alteration of the microsaccadic velocity-amplitude main sequence relationship after visual transients: Implications for models of saccade control. Journal of Neurophysiology.

[bib0011] Chan-Palay V., Asan E. (1989). Quantitation of catecholamine neurons in the locus coeruleus in human brains of normal young and older adults and in depression. Journal of Comparative Neurology.

[bib0012] Chang Y., Chen H., Barquero C., Tsai H.J., Liang W., Hsu C., Muggleton N.G., Wang C. (2023). Linking tonic and phasic pupil responses to P300 amplitude in an emotional face-word stroop task. Psychophysiology.

[bib0013] Chang Y.H., Lin C.S., Barquero C., Wang C.A. (2025). Emotional conflict affects microsaccade dynamics in the emotional face–word stroop task. Annals of the New York Academy of Sciences.

[bib0014] Chen C.C., Cho S.L., Tseng R.Y. (2013). Taiwan corpora of Chinese emotions and relevant psychophysiological data—Behavioral evaluation norm for facial expressions of professional performer. Chinese Journal of Psychology.

[bib0015] Chen J.T., Yep R., Hsu Y.F., Cherng Y.G., Wang C.A. (2021). Investigating arousal, saccade preparation, and global luminance effects on microsaccade behavior. Frontiers in Human Neuroscience.

[bib0016] Contadini-Wright C., Magami K., Mehta N., Chait M. (2023). Pupil dilation and microsaccades provide complementary insights into the dynamics of arousal and instantaneous attention during effortful listening. Journal of Neuroscience.

[bib0017] Corneil B.D., Munoz D.P. (2014). Overt responses during covert orienting. Neuron.

[bib0020] Dalmaso M., Castelli L., Scatturin P., Galfano G. (2017). Working memory load modulates microsaccadic rate. Journal of Vision.

[bib0018] Dalmaso M., Castelli L., Galfano G. (2019). Anticipation of cognitive conflict is reflected in microsaccades: Evidence from a cued-flanker task. Journal of Eye Movement Research.

[bib0019] Dalmaso M., Castelli L., Galfano G. (2020). Microsaccadic rate and pupil size dynamics in pro-/anti-saccade preparation: The impact of intermixed vs. blocked trial administration. Psychological Research.

[bib0021] De Craen A.J.M., Heeren T.J., Gussekloo J. (2003). Accuracy of the 15-item geriatric depression scale (GDS-15) in a community sample of the oldest old. International Journal of Geriatric Psychiatry.

[bib0022] del Cerro I., Martínez-Zalacaín I., Guinea-Izquierdo A., Gascón-Bayarri J., Viñas-Diez V., Urretavizcaya M., Naval-Baudin P., Aguilera C., Reñé-Ramírez R., Ferrer I., Menchón J.M., Soria V., Soriano-Mas C. (2020). Locus coeruleus connectivity alterations in late-life major depressive disorder during a visual oddball task. NeuroImage: Clinical.

[bib0023] Di Stasi L.L., McCamy M.B., Catena A., Macknik S.L., Canas J.J., Martinez-Conde S. (2013). Microsaccade and drift dynamics reflect mental fatigue. The European Journal of Neuroscience.

[bib0024] Di Stasi L.L., McCamy M.B., Pannasch S., Renner R., Catena A., Canas J.J., Velichkovsky B.M., Martinez-Conde S. (2015). Effects of driving time on microsaccadic dynamics. Experimental Brain Research.

[bib0025] Djernes J.K. (2006). Prevalence and predictors of depression in populations of elderly: A review. Acta Psychiatrica Scandinavica.

[bib0026] Drysdale A.T., Grosenick L., Downar J., Dunlop K., Mansouri F., Meng Y., Fetcho R.N., Zebley B., Oathes D.J., Etkin A., Schatzberg A.F., Sudheimer K., Keller J., Mayberg H.S., Gunning F.M., Alexopoulos G.S., Fox M.D., Pascual-Leone A., Voss H.U., Liston C. (2017). Resting-state connectivity biomarkers define neurophysiological subtypes of depression. Nature Medicine.

[bib0027] Du X., Yin M., Yuan L., Zhang G., Fan Y., Li Z., Yuan N., Lv X., Zhao X., Zou S., Deng W., Kosten T.R., Zhang X.Y. (2020). Reduction of depression-like behavior in rat model induced by ShRNA targeting norepinephrine transporter in locus coeruleus. Translational Psychiatry.

[bib0028] Edwards S.B., Ginsburgh C.L., Henkel C.K., Stein B.E. (1979). Sources of subcortical projections to the superior colliculus in the cat. The Journal of Comparative Neurology.

[bib0029] Engbert R., Kliegl R. (2003). Microsaccades uncover the orientation of covert attention. Vision Research.

[bib0030] Engbert R., Mergenthaler K. (2006). Microsaccades are triggered by low retinal image slip. Proceedings of the National Academy of Sciences.

[bib0032] Etkin A., Egner T., Peraza D.M., Kandel E.R., Hirsch J. (2006). Resolving emotional conflict: A role for the rostral anterior cingulate cortex in modulating activity in the amygdala. Neuron.

[bib0031] Etkin A., Egner T., Kalisch R. (2011). Emotional processing in anterior cingulate and medial prefrontal cortex. Trends in Cognitive Sciences.

[bib0033] Folstein M.F., Folstein S.E., McHugh P.R. (1975). “Mini-mental state”. A practical method for grading the cognitive state of patients for the clinician. Journal of Psychiatric Research.

[bib0034] Goldman P.S., Nauta W.J.H. (1976). Autoradiographic demonstration of a projection from prefrontal association cortex to the superior colliculus in the rhesus monkey. Brain Research.

[bib0035] Gorman J.M., Sloan R.P. (2000). Heart rate variability in depressive and anxiety disorders. American Heart Journal.

[bib0036] Grueschow M., Kleim B., Ruff C.C. (2020). Role of the locus coeruleus arousal system in cognitive control. Journal of Neuroendocrinology.

[bib0038] Grueschow M., Stenz N., Thörn H., Ehlert U., Breckwoldt J., Brodmann Maeder M., Exadaktylos A.K., Bingisser R., Ruff C.C., Kleim B. (2021). Real-world stress resilience is associated with the responsivity of the locus coeruleus. Nature Communications.

[bib0037] Grueschow M., Kleim B., Ruff C.C. (2022). Functional coupling of the locus coeruleus is linked to successful cognitive control. Brain Sciences.

[bib0039] Guinea-Izquierdo A., Giménez M., Martínez-Zalacaín I., del Cerro I., Canal-Noguer P., Blasco G., Gascón J., Reñé R., Rico I., Camins A., Aguilera C., Urretavizcaya M., Ferrer I., Menchón J.M., Soria V., Soriano-Mas C. (2021). Lower Locus Coeruleus MRI intensity in patients with late-life major depression. PeerJ.

[bib0040] Hafed Z.M., Clark J.J. (2002). Microsaccades as an overt measure of covert attention shifts. Vision Research.

[bib0041] Hafed Z.M., Goffart L., Krauzlis R.J. (2009). A neural mechanism for microsaccade generation in the primate superior colliculus. Science (New York, N.Y.).

[bib0042] Hafed Z.M., Lovejoy L.P., Krauzlis R.J. (2013). Superior colliculus inactivation alters the relationship between covert visual attention and microsaccades. The European Journal of Neuroscience.

[bib0043] Hermens F., Zanker J.M., Walker R. (2010). Microsaccades and preparatory set: A comparison between delayed and immediate, exogenous and endogenous pro- and anti-saccades. Experimental Brain Research.

[bib0044] Hsu T.Y., Chen J.T., Tseng P., Wang C.A. (2021). Role of the frontal eye field in human microsaccade responses: A TMS study. Biological Psychology.

[bib87] JASP Team. (2019). JASP (Version 0.10.2). [Computer software].

[bib0045] Jellinger K.A. (2023). The heterogeneity of late-life depression and its pathobiology: A brain network dysfunction disorder. Journal of Neural Transmission.

[bib0046] Klimek V., Stockmeier C., Overholser J., Meltzer H.Y., Kalka S., Dilley G., Ordway G.A. (1997). Reduced levels of norepinephrine transporters in the locus coeruleus in major depression. Journal of Neuroscience.

[bib0047] Koenig A.M., Bhalla R.K., Butters M.A. (2014). Cognitive functioning and late-life depression. Journal of the International Neuropsychological Society.

[bib0048] Krejtz K., Żurawska J., Duchowski A., Wichary S. (2020). Pupillary and microsaccadic responses to cognitive effort and emotional arousal during complex decision making. Journal of Eye Movement Research.

[bib0049] Kroenke K., Spitzer R.L., Williams J.B.W. (2002). The PHQ-15: Validity of a new measure for evaluating the severity of somatic symptoms. Psychosomatic Medicine.

[bib0050] Laubrock J., Engbert R., Kliegl R. (2005). Microsaccade dynamics during covert attention. Vision Research.

[bib0051] Lee S., Ma Y.L., Tsang A. (2011). Psychometric properties of the Chinese 15-item patient health questionnaire in the general population of Hong Kong. Journal of Psychosomatic Research.

[bib0053] Lee Y.T., Chang Y.H., Tsai H.J., Chao S.P., Chen D.Y.T., Chen J.T., Cherng Y., Wang C.A. (2024). Altered pupil light and darkness reflex and eye-blink responses in late-life depression. BMC Geriatrics.

[bib0052] Lee Y.T., Chang Y.H., Barquero C., Wu C.S., Chao S.P., Chen D.Y.T., Chen J., Cherng Y., Wang C.A. (2025). Pupil and eye blink response abnormalities during emotional conflict processing in late-life depression. Journal of Geriatric Psychiatry and Neurology.

[bib0054] Lee Y.T., Tai Y.H., Chang Y.H., Barquero C., Chao S.P., Wang C.A. (2025). Disrupted microsaccade responses in late-life depression. Scientific Reports.

[bib0055] Leichnetz G.R., Spencer R.F., Hardy S.G.P., Astruc J. (1981). The prefrontal corticotectal projection in the monkey; an anterograde and retrograde horseradish peroxidase study. Neuroscience.

[bib0056] Li L.L., Feng X., Zhou Z., Zhang H., Shi Q., Lei Z., Shen P., Yang Q., Zhao B., Chen S., Li L.L., Zhang Y., Wen P., Lu Z., Li X., Xu F., Wang L. (2018). Stress accelerates defensive responses to looming in mice and involves a locus coeruleus-superior colliculus projection. Current Biology.

[bib0057] Liao Y., Yeh T., Ko H., Luoh C., Lu F. (1995). Geriatric depression scale–validity and reliabilty of the Chinese-translated version: A preliminary study. The Changhua Journal of Medicine.

[bib0058] Liu K.Y., Kievit R.A., Tsvetanov K.A., Betts M.J., Düzel E., Rowe J.B., Tyler L.K., Brayne C., Bullmore E.T., Calder A.C., Cusack R., Dalgleish T., Duncan J., Henson R.N., Matthews F.E., Marslen-Wilson W.D., Shafto M.A., Campbell K., Cheung T., Hämmerer D. (2020). Noradrenergic-dependent functions are associated with age-related locus coeruleus signal intensity differences. Nature Communications.

[bib0059] Llorca-Torralba M., Camarena-Delgado C., Suárez-Pereira I., Bravo L., Mariscal P., Garcia-Partida J.A., López-Martín C., Wei H., Pertovaara A., Mico J.A., Berrocoso E. (2022). Pain and depression comorbidity causes asymmetric plasticity in the locus coeruleus neurons. Brain: A Journal of Neurology.

[bib0060] Lockwood K.A., Alexopoulos G.S., Van Gorp W.G. (2002). Executive dysfunction in geriatric depression. American Journal of Psychiatry.

[bib0061] Malevich T., Buonocore A., Hafed Z.M. (2021). Dependence of the stimulus-driven microsaccade rate signature in rhesus macaque monkeys on visual stimulus size and polarity. Journal of Neurophysiology.

[bib0063] Mather M., Harley C.W. (2016). The Locus Coeruleus: Essential for maintaining cognitive function and the aging brain. Trends in Cognitive Sciences.

[bib0062] Mather M., Clewett D., Sakaki M., Harley C.W. (2016). Norepinephrine ignites local hotspots of neuronal excitation: How arousal amplifies selectivity in perception and memory. The Behavioral and Brain Sciences.

[bib0064] Nasreddine Z.S., Phillips N.A., Bédirian V., Charbonneau S., Whitehead V., Collin I., Cummings J.L., Chertkow H. (2005). The Montreal Cognitive Assessment, MoCA: A brief screening tool for mild cognitive impairment. Journal of the American Geriatrics Society.

[bib0065] Penninx B.W.J.H., Milaneschi Y., Lamers F., Vogelzangs N. (2013). Understanding the somatic consequences of depression: Biological mechanisms and the role of depression symptom profile. BMC Medicine.

[bib0066] Poe G.R., Foote S., Eschenko O., Johansen J.P., Bouret S., Aston-Jones G., Harley C.W., Manahan-Vaughan D., Weinshenker D., Valentino R. (2020). Locus coeruleus: A new look at the blue spot. Nature Reviews Neuroscience.

[bib85] R Core Team (2020). R: A language and environment for statistical computing. R Foundation for Statistical Computing.

[bib0067] Rajtar-Zembaty A., Sałakowski A., Rajtar-Zembaty J., Starowicz-Filip A. (2017). Executive dysfunction in late-life depression. Psychiatria Polska.

[bib86] Rstudio Team (2019). RStudio: Integrated development for.

[bib0068] Siegenthaler E., Costela F.M., McCamy M.B., Di Stasi L.L., Otero-Millan J., Sonderegger A., Groner R., Macknik S., Martinez-Conde S. (2014). Task difficulty in mental arithmetic affects microsaccadic rates and magnitudes. The European Journal of Neuroscience.

[bib0069] Soncin S., Brien D.C., Coe B.C., Marin A., Munoz D.P. (2016). Contrasting emotion processing and executive functioning in attention-deficit/hyperactivity disorder and bipolar disorder. Behavioral Neuroscience.

[bib0070] Szymkowicz S.M., Gerlach A.R., Homiack D., Taylor W.D. (2023). Biological factors influencing depression in later life: Role of aging processes and treatment implications. Translational Psychiatry.

[bib0071] Tang T., Jiang J., Tang X. (2022). Prevalence of depression among older adults living in care homes in China: A systematic review and meta-analysis. International Journal of Nursing Studies.

[bib0072] Tsai C.F., Lee W.J., Wang S.J., Shia B.C., Nasreddine Z., Fuh J.L. (2012). Psychometrics of the Montreal Cognitive Assessment (MoCA) and its subscales: Validation of the Taiwanese version of the MoCA and an item response theory analysis. International Psychogeriatrics.

[bib0073] Tsopelas C., Stewart R., Savva G.M., Brayne C., Ince P., Thomas A., Matthews F.E. (2011). Neuropathological correlates of late-life depression in older people. British Journal of Psychiatry.

[bib0074] Valsecchi M., Turatto M. (2009). Microsaccadic responses in a bimodal oddball task. Psychological Research.

[bib0075] Van Damme A., Declercq T., Lemey L., Tandt H., Petrovic M. (2018). Late-life depression: Issues for the general practitioner. International Journal of General Medicine.

[bib0078] Wang C.A., Munoz D.P. (2021). Differentiating global luminance, arousal and cognitive signals on pupil size and microsaccades. European Journal of Neuroscience.

[bib0079] Wang Chin-An, Munoz D.P., Papesh M., Goldinger S. (2024). Modern Pupillometry: Cognition, Neuroscience, and Practical Applications.

[bib0076] Wang C.A., Baird T., Huang J., Coutinho J.D., Brien D.C., Munoz D.P. (2018). Arousal effects on pupil size, heart rate, and skin conductance in an emotional face task. Frontiers in Neurology.

[bib0077] Wang C.A., Huang J., Brien D.C., Munoz D.P. (2020). Saliency and priority modulation in a pop-out paradigm: Pupil size and microsaccades. Biological Psychology.

[bib0080] Watanabe M., Matsuo Y., Zha L., Munoz D.P., Kobayashi Y. (2013). Fixational saccades reflect volitional action preparation. Journal of Neurophysiology.

[bib0081] White B.J., Munoz D.P., Liversedge S., Gilchrist I., Everling S. (2011). Oxford Handbook of Eye Movements.

[bib0082] Willenbockel V., Sadr J., Fiset D., Horne G.O., Gosselin F., Tanaka J.W. (2010). Controlling low-level image properties: The SHINE toolbox. Behavior Research Methods.

[bib0083] World Medical Association (2001). World Medical Association Declaration of Helsinki. Ethical principles for medical research involving human subjects. Bulletin of the World Health Organization.

[bib0084] Yep R., Soncin S., Brien D.C., Coe B.C., Marin A., Munoz D.P. (2018). Using an emotional saccade task to characterize executive functioning and emotion processing in attention-deficit hyperactivity disorder and bipolar disorder. Brain and Cognition.

