# Supplementary material for: Altered oculomotor signatures of emotional conflict and valence processing in late-life depression
Source: Int J Clin Health Psychol. 2026 Jul 31;26(2):100711. doi: 10.1016/j.ijchp.2026.100711 (PMC13452990; doi:10.1016/j.ijchp.2026.100711)
Supplement: Supplementary file 1 [file mmc1.pdf]

Supplementary Table 1. Medication use at the time of assessment by diagnostic group

Group	CTRL	LLD
Medication class	N = 27	N = 26
SSRI	1	14
SNRI	0	1
NDRI	0	3
TCA	2	2
MAOI	0	0
Anti-cholinergic	0	0
α-blocker	4	1
β-blocker	5	9
Anti-histamine	0	0
Benzodiazepine	12	20

Values are numbers and percentages of participants. Categories are not mutually exclusive because participants could be taking medications from more than one class. SSRI, selective serotonin reuptake inhibitor; SNRI, serotonin–norepinephrine reuptake inhibitor; NDRI, norepinephrine–dopamine reuptake inhibitor; TCA, tricyclic antidepressant.

Supplementary Table 2. Medication-adjusted hierarchical linear mixed-effects analyses of primary microsaccade outcomes

**Rate (face-epoch)**	**AIC**	**BIC**	**$\chi 2$**	**df**	**p**	
Covariate model	173.0	199.6				
Group	170.3	199.6	4.70	1	0.030	*
Group + Congruency	163.2	195.2	9.06	1	0.003	**
Group x Congruency	163.6	198.2	1.65	1	0.199	

Rate ~ Group + Congruency + Medication Used + 1|Subj

	**Beta**	**Std. Error**	**df**	**t**	**p**	
Intercept	2.89	0.26	44	11.21	0.000	***
Group	0.39	0.19	44	2.02	0.049	*
Congruency	-0.05	0.02	52	-3.11	0.003	**
SSRI	0.66	0.42	44	1.57	0.124	
SNRI	0.98	1.07	44	0.91	0.367	
NDRI	0.58	0.68	44	0.86	0.395	
TCA	1.04	0.57	44	1.85	0.071	
Alpha-blocker	0.09	0.49	44	0.18	0.861	
Beta-blocker	-0.36	0.34	44	-1.05	0.302	
BZD	-0.26	0.33	44	-0.78	0.441	

**Ratio (face-epoch)**	**AIC**	**BIC**	**$\chi 2$**	**df**	**p**	
Covariate model	2170.6	2197.2				
Group	2168.6	2197.8	4.04	1	0.044	*
Group + Congruency	2167.2	2199.1	3.39	1	0.066	

Peak-velocity-to-amplitude ratio ~ Group + Medication Used + 1|Subj

	**Beta**	**Std. Error**	**df**	**t**	**p**	
Intercept	129.73	2.55	44	50.84	<2e-16	***
Group	-3.55	1.90	44	-1.87	0.069	
SSRI	1.39	4.13	44	0.34	0.739	
SNRI	3.57	10.59	44	0.34	0.738	
NDRI	-2.56	6.70	44	-0.38	0.705	
TCA	6.25	5.59	44	1.12	0.269	
Alpha-blocker	8.08	4.81	44	1.68	0.100	
Beta-blocker	-5.81	3.38	44	-1.72	0.093	
BZD	-0.31	3.28	44	-0.09	0.926	

Amp (response epoch)	AIC	BIC	χ2	df	p	
Covariate model	-224.3	-197.6				
Group	-222.9	-193.6	0.61	1	0.436	
Congruency	-226.6	-197.3	4.36	1	0.037	*

Amp ~ Congruency + Medication Used + 1|Subj

	Beta	Std. Error	df	t	p	
Intercept	0.81	0.03	45	27.23	<2e-16	***
Congruency	-0.01	0.00	52	-2.11	0.040	*
SSRI	-0.01	0.04	45	-0.13	0.899	
SNRI	-0.11	0.14	45	-0.80	0.428	
NDRI	0.04	0.08	45	0.46	0.651	
TCA	-0.11	0.07	45	-1.46	0.151	
Alpha-blocker	-0.04	0.06	45	-0.63	0.534	
Beta-blocker	0.06	0.05	45	1.34	0.189	
BZD	-0.06	0.04	45	-1.39	0.172	

Rate (face epoch)	AIC	BIC	χ2	df	p	
Covariate model	241.5	268.1				
Group	237.7	267.0	5.80	1	0.016	*
Group + Valence	233.3	265.3	6.43	1	0.011	*
Group x Valence	235.3	269.9	0.00	1	0.994	

Rate ~ Group + Valence + Medication Used + 1|Subj

	Beta	Std. Error	df	t	p	
Intercept	2.81	0.26	44	10.96	0.000	***
Group	0.43	0.19	44	2.26	0.029	*
Valence	0.09	0.03	52	2.59	0.013	*
SSRI	0.67	0.42	44	1.61	0.116	
SNRI	1.08	1.06	44	1.01	0.317	
NDRI	0.62	0.67	44	0.93	0.360	
TCA	1.05	0.56	44	1.87	0.068	
Alpha-blocker	0.01	0.48	44	0.02	0.985	
Beta-blocker	-0.43	0.34	44	-1.26	0.215	

BZD	-0.17	0.33	44	-0.51	0.613

Medication-class indicators included SSRI, SNRI, NDRI, TCA, α-blocker, β-blocker, and benzodiazepine use, coded as 1 or 0. Models included a participant-specific random intercept. Nested models differing in fixed effects were compared using maximum-likelihood estimation. These analyses were conducted as robustness checks; individual medication coefficients were not interpreted as estimates of causal medication effects because medication exposure was nonrandom and several medication classes had sparse representation. SSRI, selective serotonin reuptake inhibitor; SNRI, serotonin–norepinephrine reuptake inhibitor; NDRI, norepinephrine–dopamine reuptake inhibitor; TCA, tricyclic antidepressant. BZD, benzodiazepine.

Supplementary Table 3. Univariate ROC–AUC values for individual eye-tracking indices differentiating late-life depression from healthy controls

Feature	Description	ROC–AUC
incon_con_rate	Microsaccade rate contrast (incongruent − congruent)	0.595
incon_con_amp	Microsaccade amplitude contrast (incongruent − congruent)	0.385
incon_con_vel	Microsaccade peak velocity contrast (incongruent − congruent)	0.423
incon_con_RT	Reaction time contrast (incongruent − congruent)	0.648
fear_happy_rate	Microsaccade rate contrast (fearful − happy)	0.501
fear_happy_amp	Microsaccade amplitude contrast (fearful − happy)	0.513
fear_happy_vel	Microsaccade peak velocity contrast (fearful − happy)	0.504
fear_happy_RT	Reaction time contrast (fearful − happy)	0.38
incon_con_com_1	Pupil component 1 score (incongruent − congruent)	0.568
incon_con_com_2	Pupil component 2 score (incongruent − congruent)	0.521
fear_happy_com_1	Pupil component 1 score (fearful − happy)	0.472
fear_happy_com_2	Pupil component 2 score (fearful − happy)	0.352
incon_con_blink	Eye blink rate contrast (incongruent − congruent)	0.593
fear_happy_blink	Eye blink rate contrast (fearful − happy)	0.47

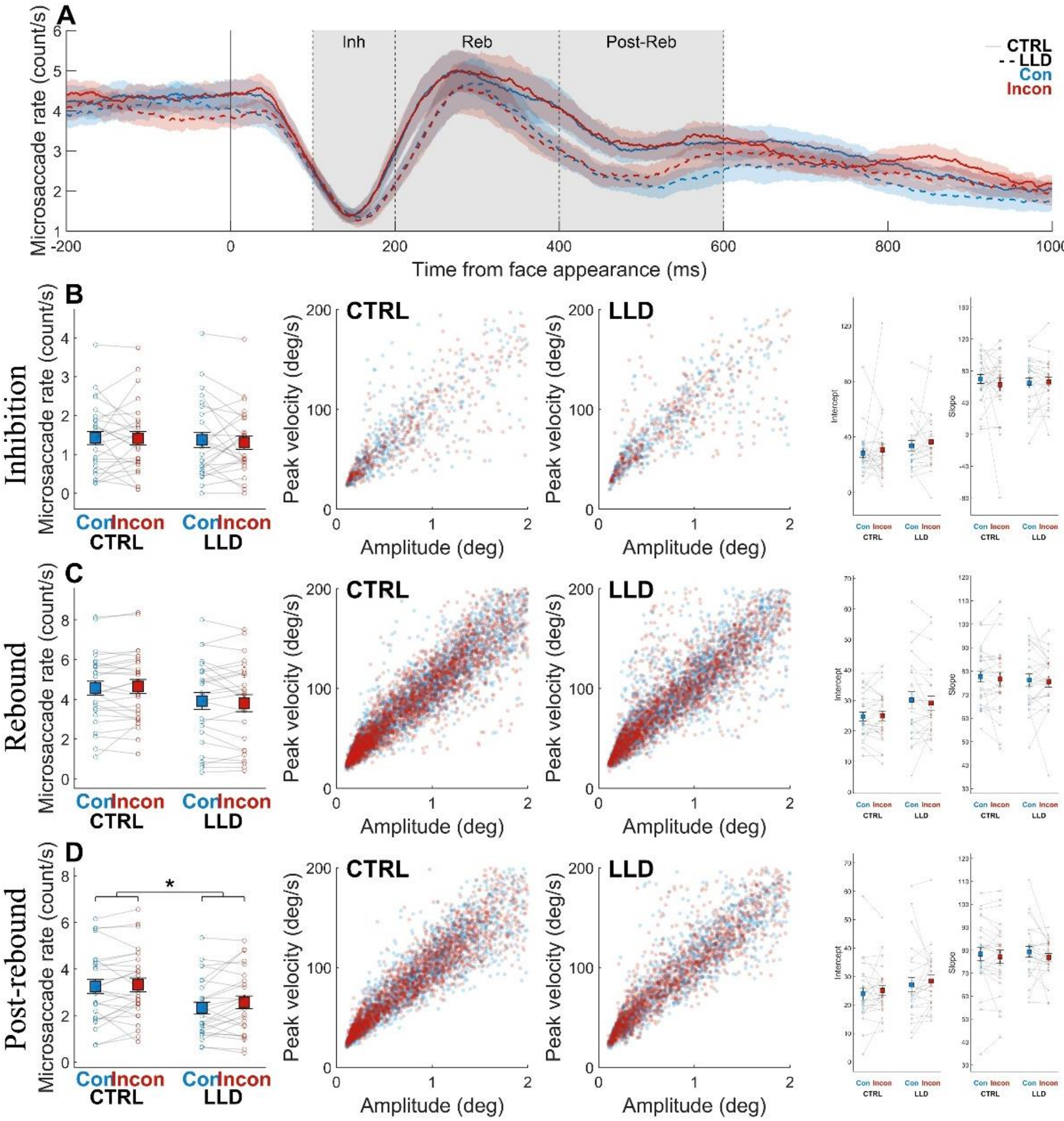

Supplementary Figure 1. Phase-specific effects of emotional congruency on face-evoked microsaccade rate and main-sequence parameters. (A) Microsaccade-rate dynamics time-locked to face appearance, with three analysis periods: inhibition (Inh, 100–200 ms), rebound (Reb, 200–400 ms), and post-rebound (Post-Reb, 400–600 ms). **(B–D)** Mean microsaccade rates, microsaccade main-sequence relationships, and participant-level main-sequence intercepts and slopes during the inhibition, rebound, and post-rebound periods, respectively. Shaded colored regions surrounding the rate curves represent ± standard error of the mean across participants. Filled squares and error bars represent the group mean ± standard error, open circles represent individual-participant means, and gray lines connect the two conditions within each participant. Colored points in the main-sequence plots represent individual microsaccades pooled across

participants. During the post-rebound period, microsaccade rate was nominally lower in LLD than in CTRL participants, $F(1,51) = 4.81$, $p = .033$, $q = .297$, $\eta p^2 = .09$; however, this effect did not survive FDR correction. No rate or main-sequence effect survived FDR correction within the corresponding analysis families. No significant group, congruency, or interaction effects were observed for rate during the inhibition or rebound periods, and no significant effects on main-sequence intercept or slope were observed during any period. Gray areas represent the periods selected for analysis. Con, congruent condition; Incon, incongruent condition; Inh, inhibition; Reb, rebound; Post-Reb, post-rebound; CTRL, healthy older adults; LLD, individuals with late-life depression. * indicates a nominal effect at the uncorrected level ($p < .05$); this effect did not survive FDR correction.

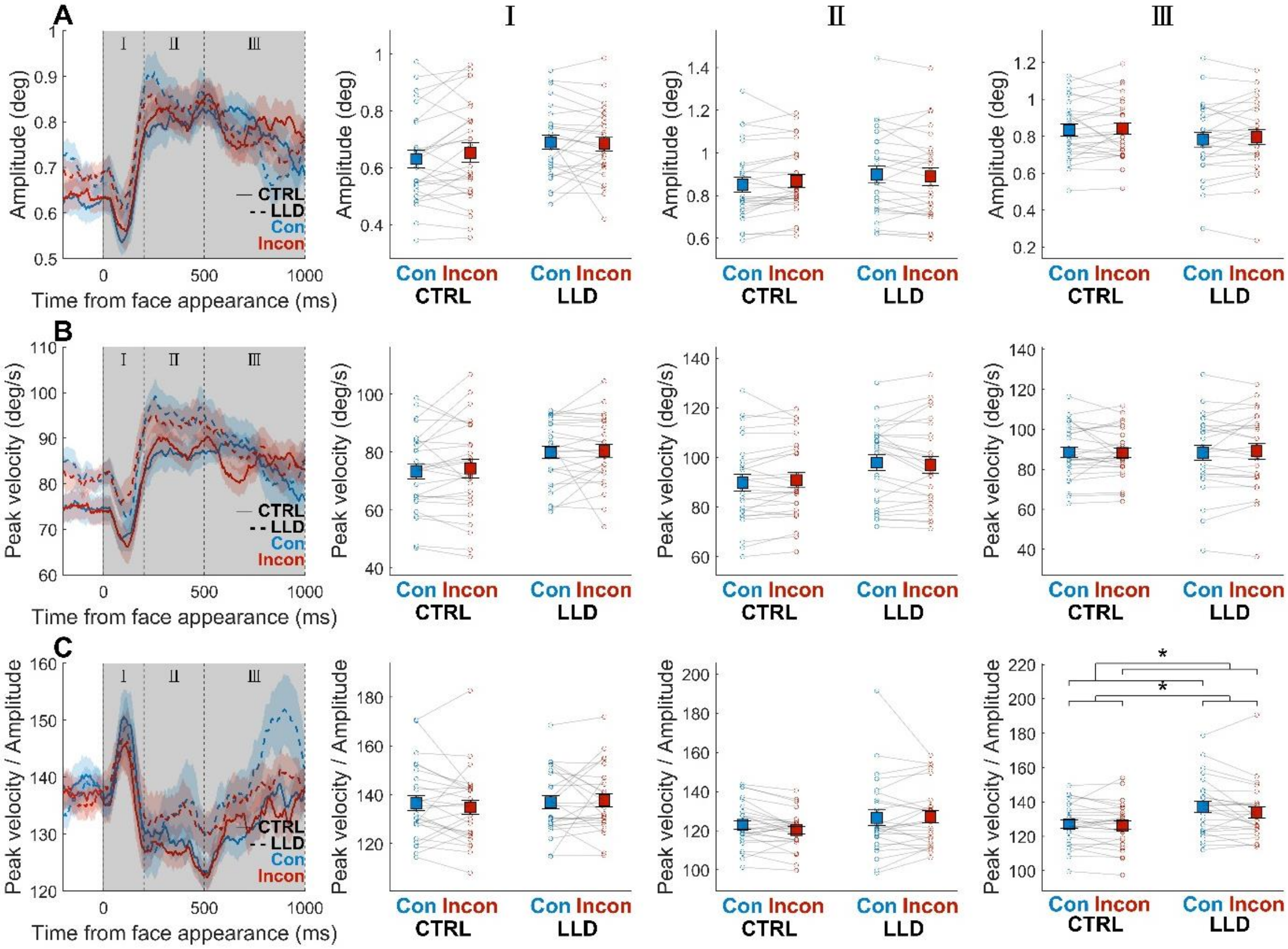

Supplementary Figure 2. Time-resolved effects of emotional congruency on microsaccade kinematics following face onset. (A) Microsaccade amplitude, **(B)** peak velocity, and **(C)** peak-velocity-to-amplitude ratio shown as time-resolved dynamics and participant-level mean values for three face-locked intervals: I, 0–200 ms; II, 200–500 ms; and III, 500–1000 ms. Shaded colored regions surrounding microsaccade dynamics curves represent ± standard error of the mean across participants. Filled squares and error bars represent the group mean ± standard error, open circles represent individual-participant means, and gray lines connect the two conditions within each participant. During interval III, nominal effects were observed for the peak-velocity-to-amplitude ratio, which was higher in LLD than in CTRL participants, $p = .026$, $q = .621$, and lower in incongruent than congruent trials across groups, $p = .049$, $q = .621$. Neither effect survived FDR correction, and no interval-specific kinematic effect survived correction within the corresponding analysis family. No significant group, congruency, or interaction effects were observed for amplitude or peak velocity in any interval. The gray area represents the epoch

selected for analyses. Con, congruent condition; Incon, incongruent condition; CTRL, healthy older adults; LLD, individuals with late-life depression. * indicates a nominal effect at the uncorrected level ($p < .05$); this effect did not survive FDR correction.

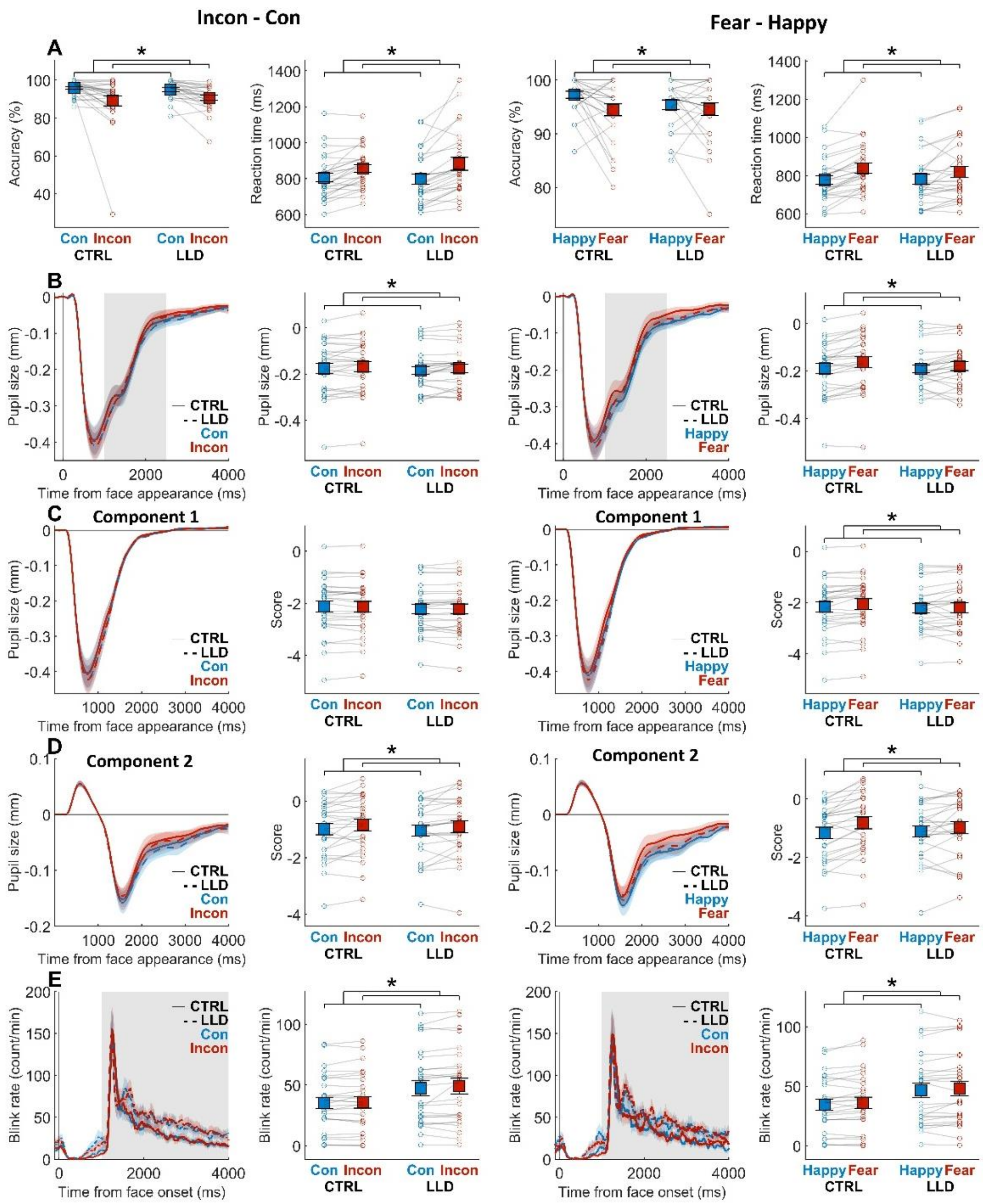

Supplementary Figure 3. Emotional congruency and valence effects on behavioral and pupillary responses. (A) Accuracy and correct reaction time in different conditions and experimental groups. **(B)** Pupil dynamics following face-word presentation and mean pupil sizes in the pupil epoch (1000 to 2500 ms) in different conditions and experimental groups. **(C)** PCA component 1 dynamics and PCA component 1 scores in different conditions and experimental groups. **(D)** PCA component 2 dynamics and PCA component 2 scores in different conditions and experimental groups. **(E)** Eye-blink dynamics following face-word presentation and mean eye-blink rates (1000 to 4000 ms) in different conditions and experimental groups. The color-

filled squares and error-bars represent mean value ± standard error (across participants) for each condition, and the small circles represent mean value for each subject. The gray area represents the epoch selected for analyses. The shaded colored regions surrounding the response represent ± standard error range for different conditions. Con: congruent condition. Incon: incongruent condition. Happy: happy condition. Fear: fear condition. Component 1: first component. Component 2: second component. CTRL: healthy older adults. LLD: late-life depression patients. PCA: principal component analysis. * indicates a statistically significant difference.

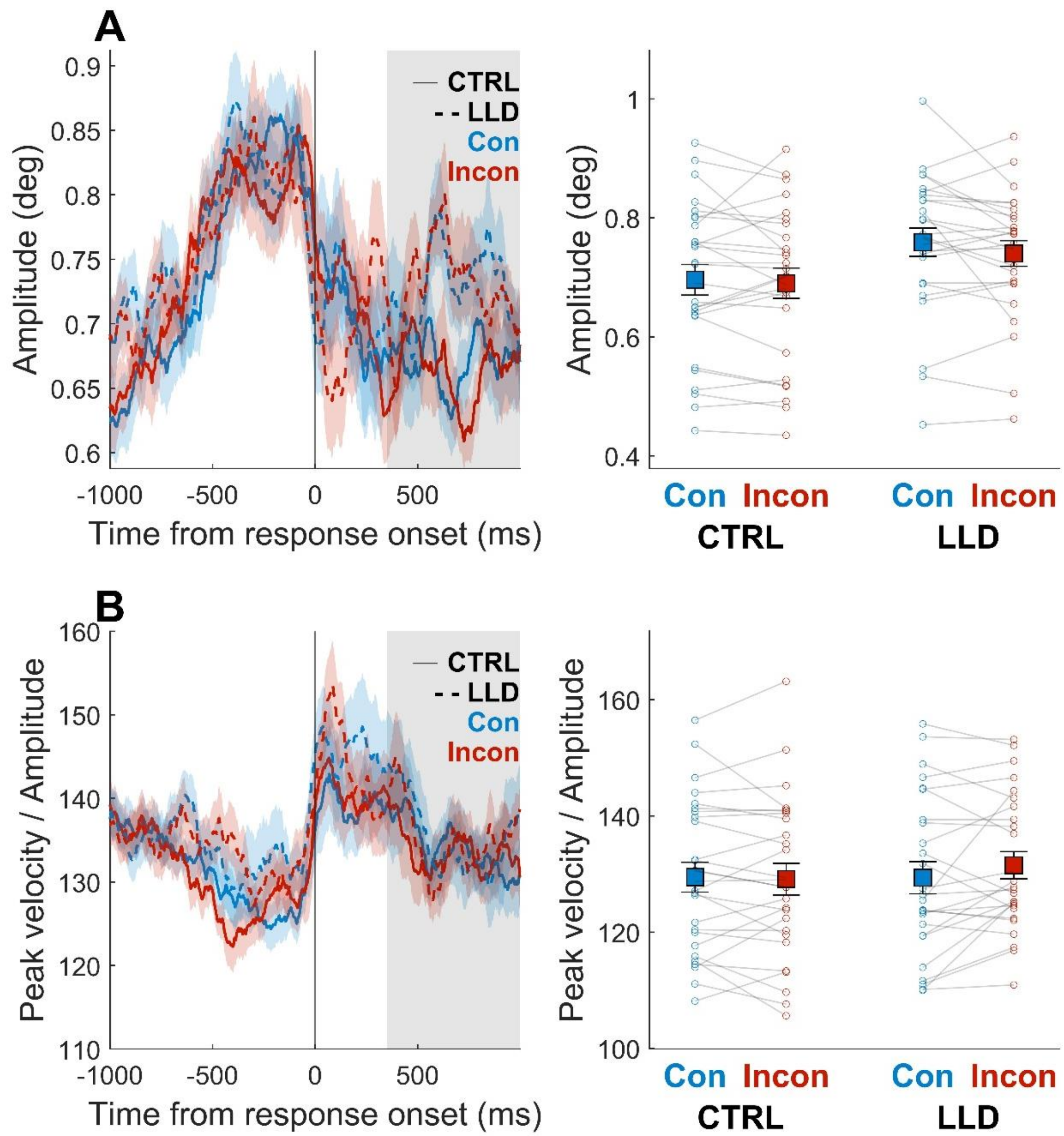

Supplementary Figure 4. Post-response microsaccade kinematics as a function of emotional congruency. Response-locked time courses and participant-level summaries of (A) microsaccade amplitude and **(B)** the peak-velocity-to-amplitude ratio in CTRL and LLD participants during congruent and incongruent trials. The vertical line indicates manual-response onset, and the gray region indicates the 350–1000-ms post-response interval used for statistical analysis. In this secondary analysis, no post-response amplitude or peak-velocity-to-amplitude-ratio effect survived FDR correction. The group effect on amplitude was not significant, $F(1,51) = 2.99$, $p = .090$, $q = .260$, $\eta_p^2 = .06$; all corrected q values were > .26. Shaded bands represent ± SEM. Squares and error bars represent group means ± SE; open circles represent individual-

participant means, and gray lines connect conditions within participants. CTRL, healthy older controls; LLD, late-life depression; Con, congruent; Incon, incongruent.

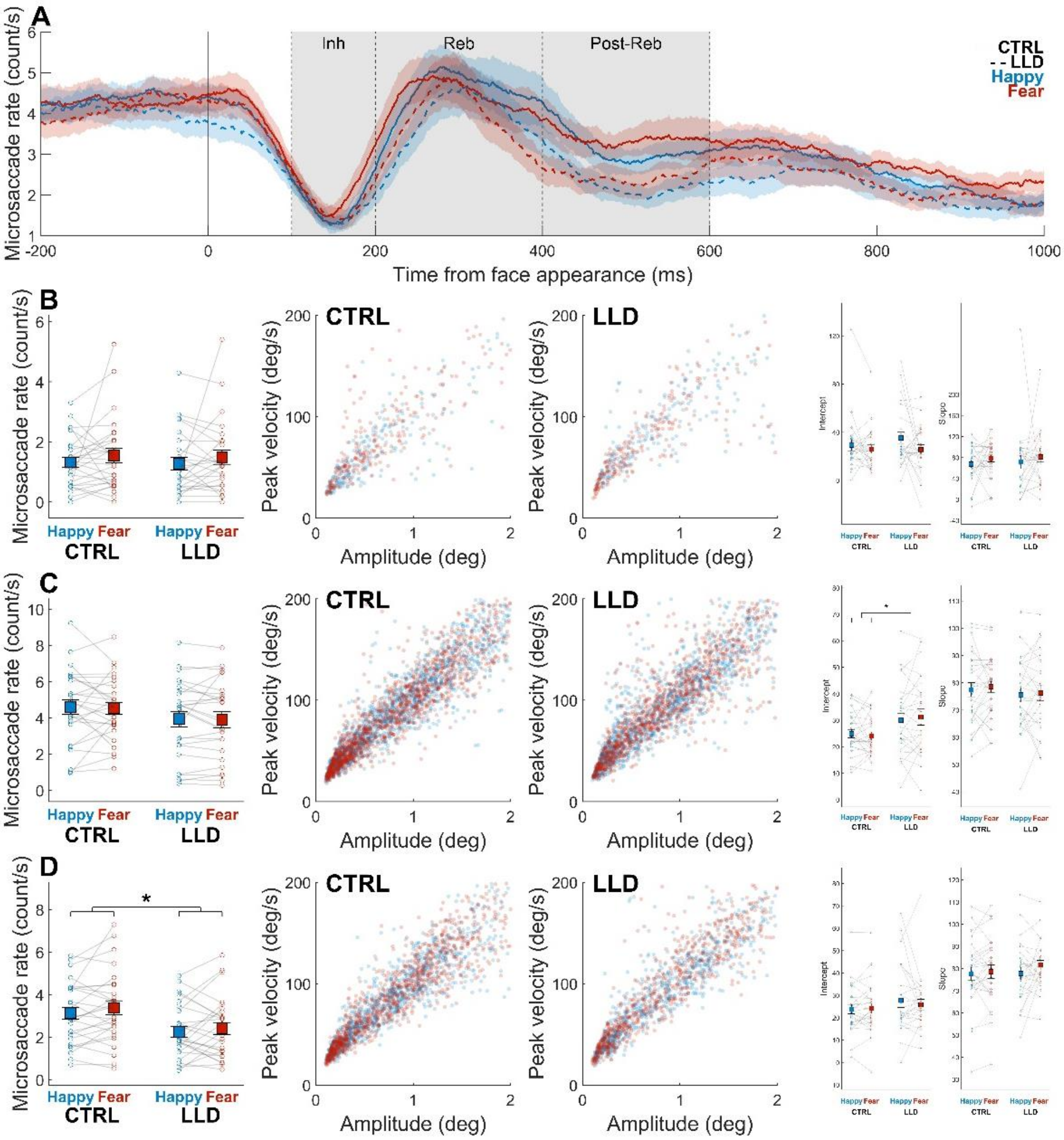

Supplementary Figure 5. Phase-specific effects of emotional valence on face-evoked microsaccade rate and main-sequence parameters. (A) Microsaccade-rate dynamics time-locked to face appearance, with three analysis periods: inhibition (Inh, 100–200 ms), rebound (Reb, 200–400 ms), and post-rebound (Post-Reb, 400–600 ms). **(B–D)** Mean microsaccade rates, microsaccade main-sequence relationships, and participant-level main-sequence intercepts and slopes during the inhibition, rebound, and post-rebound periods, respectively. Shaded colored regions surrounding the rate curves represent ± standard error of the mean across participants. Filled squares and error bars represent the group mean ± standard error, open circles represent individual-participant means, and gray lines connect the two conditions within each participant. Colored points in the main-sequence plots represent individual microsaccades pooled across

participants. During the rebound period, the main-sequence intercept was nominally higher in LLD than in CTRL participants, $F(1,51) = 4.22$, $p = .045$, $q = .516$, $\eta p^2 = .08$. During the post-rebound period, microsaccade rate was nominally lower in LLD than in CTRL participants, $F(1,51) = 5.80$, $p = .020$, $q = .180$, $\eta p^2 = .10$. Neither effect survived FDR correction, and no phase-specific rate or main-sequence effect survived correction within the corresponding analysis family. Gray areas represent the periods selected for analysis. Happy, happy condition; Fear, fear condition; Inh, inhibition; Reb, rebound; Post-Reb, post-rebound; CTRL, healthy older adults; LLD, individuals with late-life depression. * indicates a nominal effect at the uncorrected level ($p < .05$); this effect did not survive FDR correction.

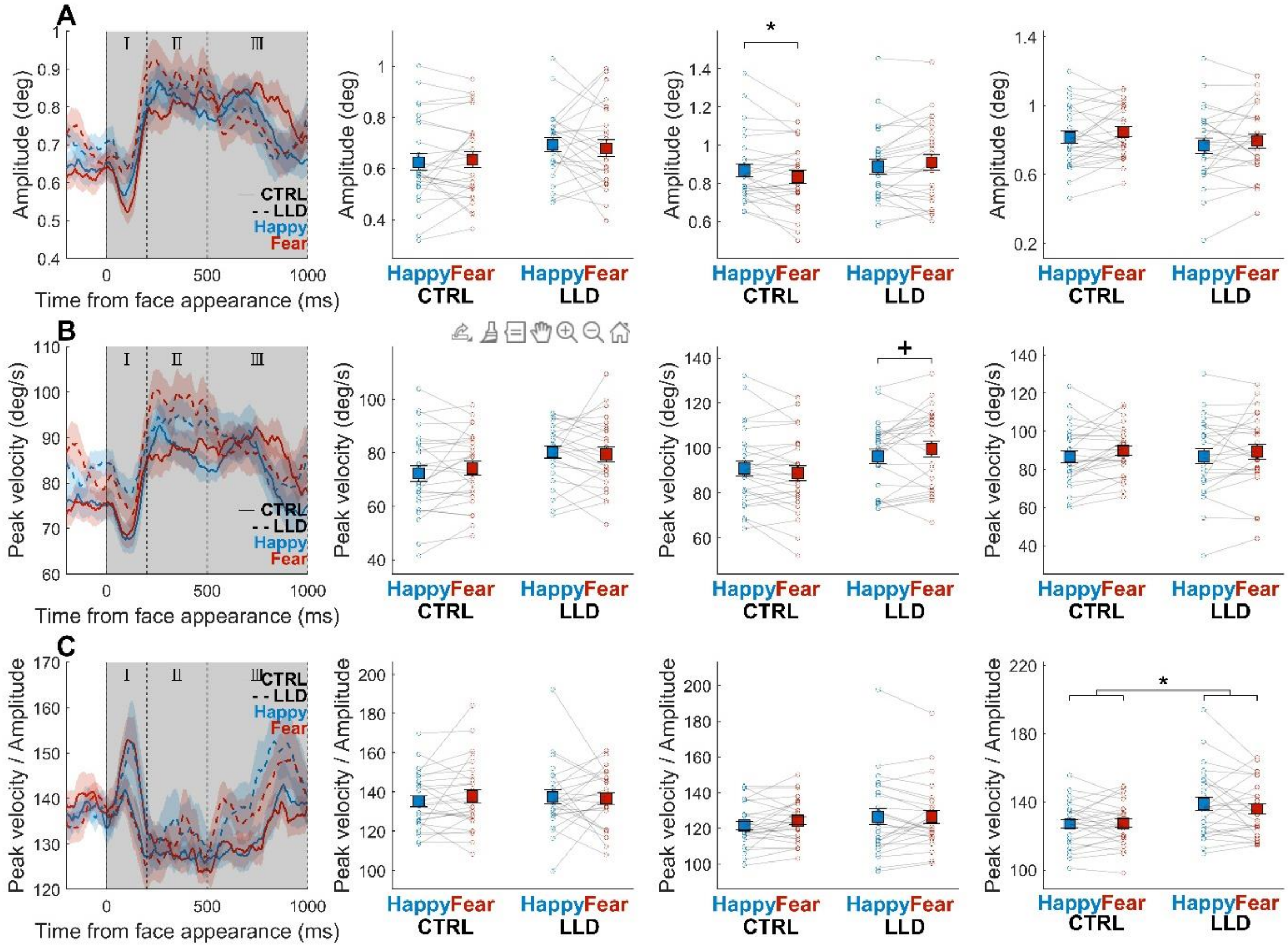

Supplementary Figure 6. Time-resolved effects of emotional valence on microsaccade kinematics following face onset. (A) Microsaccade amplitude, **(B)** peak velocity, and **(C)** peak-velocity-to-amplitude ratio shown as time-resolved dynamics and participant-level mean values for three face-locked intervals: I, 0–200 ms; II, 200–500 ms; and III, 500–1000 ms. Shaded colored regions surrounding microsaccade dynamics curves represent ± standard error of the mean across participants. Filled squares and error bars represent the group mean ± standard error, open circles represent individual-participant means, and gray lines connect the two conditions within each participant. During interval II, nominal group × valence interactions were observed for amplitude, p = .018, q = .198, and peak velocity, p = .022, q = .198. During interval III, a nominal group effect was observed for the peak-velocity-to-amplitude ratio, with higher ratios in LLD than in CTRL participants, p = .015, q = .198. None of these effects survived FDR correction. No other significant group, valence, or interaction effects were observed across the three intervals. The gray area represents the epoch selected for analyses. Happy, happy

condition; Fear, fearful condition; CTRL, healthy older adults; LLD, individuals with late-life depression. * indicates a nominal effect at the uncorrected level ($p < .05$); this effect did not survive FDR correction. + indicates a trend-level effect at the uncorrected level ($.05 \leq p < .10$); this effect did not survive FDR correction.

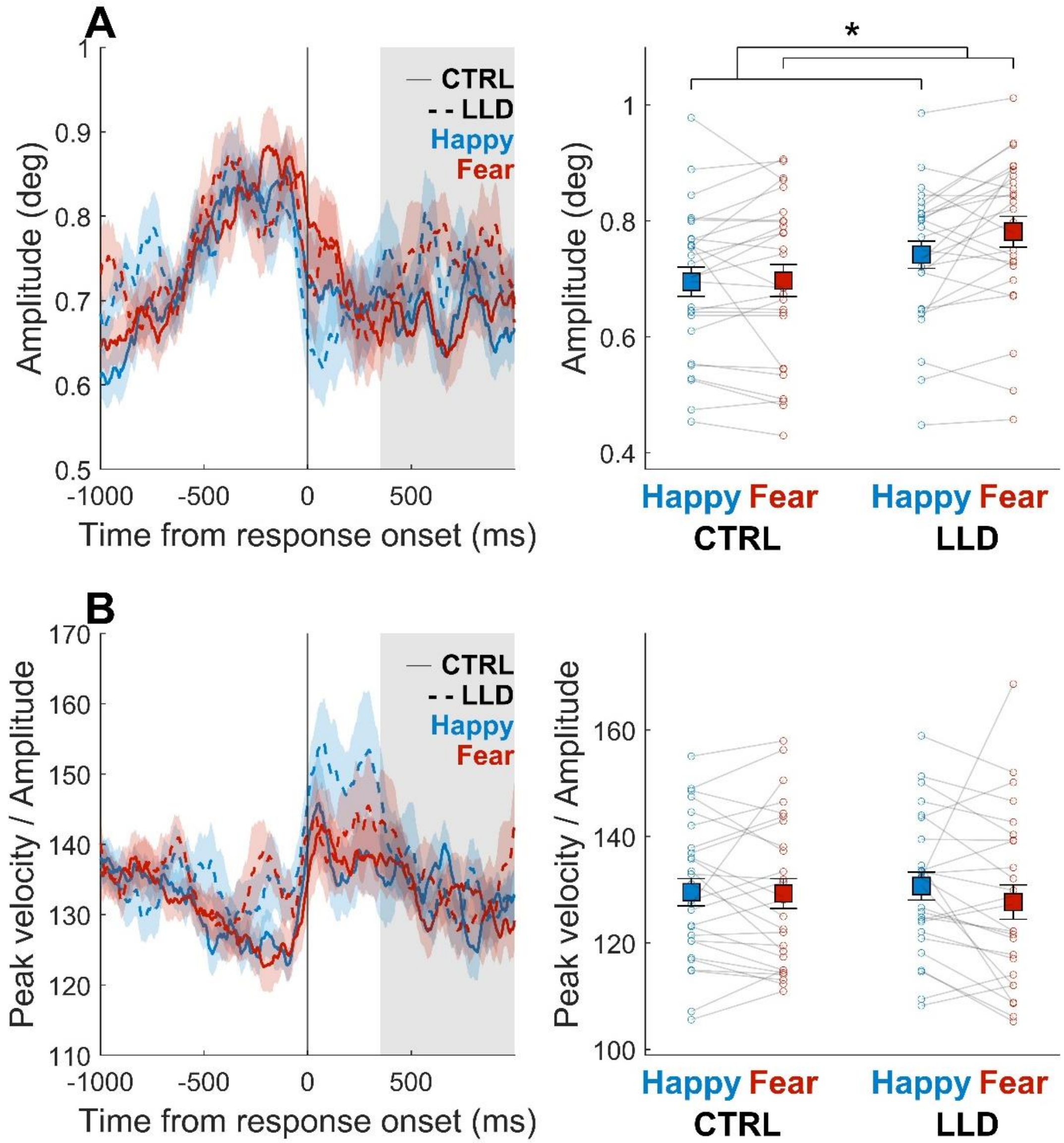

Supplementary Figure 7. Post-response microsaccade kinematics as a function of emotional valence. Response-locked time courses and participant-level summaries of (A) microsaccade amplitude and **(B)** the peak-velocity-to-amplitude ratio in CTRL and LLD participants during happy and fear trials. The vertical line indicates manual-response onset, and the gray region indicates the 350–1000-ms post-response interval used for statistical analysis. During the 350–1000-ms post-response interval, amplitude showed a nominal valence effect, with larger amplitudes for fear than happy trials, $F(1,51) = 4.61$, $p = .037$, $q = .126$, $\eta_p^2 = .08$. The group effect, $p = .063$, $q = .126$, and group × valence interaction, $p = .058$, $q = .126$, were not significant after correction. No amplitude or peak-velocity-to-amplitude-ratio effect survived

FDR correction. Shaded bands represent ± SEM. Squares and error bars represent group means ± SE; open circles represent individual-participant means, and gray lines connect conditions within participants. CTRL, healthy older controls; LLD, late-life depression. * indicates a nominal effect at the uncorrected level ($p < .05$); this effect did not survive FDR correction.
